# Co-design and content validity of the movement measurement in the early years (MoveMEY) tool for assessing movement behaviour of pre-school aged children

**DOI:** 10.1186/s12966-023-01486-2

**Published:** 2023-08-04

**Authors:** Sophie M. Phillips, Carolyn Summerbell, Kathryn R. Hesketh, Sonia Saxena, Frances C. Hillier-Brown

**Affiliations:** 1 Department of Sport and Exercise Sciences, Durham University, Durham City, UK; 2 The Centre for Translational Research in Public Health (Fuse), Newcastle Upon Tyne, UK; 3 MRC Epidemiology Unit, University of Cambridge, Cambridge, UK; 4 Population Policy & Practice Research and Teaching Department, UCL Great Ormond Street Institute of Child Health, London, UK; 5 School of Public Health, Imperial College London, London, UK; 6 Population Health Sciences Institute, Newcastle University, Newcastle upon Tyne, UK; 7 Human Nutrition Research Centre, Newcastle University, Newcastle upon Tyne, UK; 8 Newcastle University Centre of Research Excellence in Healthier Lives, Newcastle University, Newcastle upon Tyne, UK

**Keywords:** Physical activity, Sedentary behaviour, Sleep, Movement behaviour, Measurement, Diary, Pre-school, Co-design, Development, Content validity

## Abstract

**Background:**

Movement behaviours (physical activity, sedentary behaviour, and sleep) are important for pre-school children’s health and development. Currently, no tools with appropriate content validity exist that concurrently capture these movement behaviours in young children. The aim of this study was to co-design and assess the content validity of a novel tool to concurrently measure movement behaviours in pre-school aged children (aged 3–4 years).

**Methods:**

We followed four distinct steps to develop and assess the content validity of Movement Measurement in the Early Years (MoveMEY): (1) We conducted an extensive literature search, to identify pre-existing proxy measurement tools (questionnaires and diaries) to inform the design of a novel tool, which aimed to effectively capture movement behaviour guidelines of pre-school aged children. (2) We facilitated focus group discussions with parents and carers of pre-school aged children (n = 11) and (3) a qualitative survey with free text responses was completed by topic relevant researchers (n = 6), to co-design the measurement tool. (4) We assessed the content validity of the developed tool, MoveMEY, through interviews with parents of pre-school aged children (n = 12) following piloting of the tool.

**Results:**

We developed an initial version of MoveMEY based on the format of an existing questionnaire and by mapping the content of questions to the guidelines. Co-design of MoveMEY resulted in changes to the format (e.g. short questionnaire to a seven-day diary) and content (e.g. inclusion of ‘general information’ questions on illness, disabilities and sleep disturbances; question on screen time before bed). Content validity assessment demonstrated that the items of MoveMEY were relevant and comprehensive for the assessment of children’s movement behaviours. MoveMEY was felt to be comprehensible, however, parental suggestions were implemented to finalise and improve MoveMEY (e.g. adding examples to questions aiming to detect moderate to vigorous physical activity).

**Conclusion:**

MoveMEY is the first co-designed measurement tool that has relevance for assessing the movement behaviour guidelines of pre-school aged children. Parent/carer and topic relevant researcher involvement throughout the development process resulted in a seven-day daily reported activity diary that is comprehensive of children’s movement behaviours and comprehensible to parents and carers.

**Supplementary Information:**

The online version contains supplementary material available at 10.1186/s12966-023-01486-2.

## Background

Physical activity (PA), sedentary behaviour (SB), and sleep are three important movement behaviours associated with health and developmental outcomes of children in their early years [1–3]. Previously these behaviours have been examined independently but recent evidence suggests that movement behaviours may interact to influence health [4]. As such, they should be examined concurrently rather than in isolation. The importance of an integrated approach to promote healthy movement behaviours was highlighted by the World Health Organization (WHO) in their 2017 Report on Ending Childhood Obesity [5]. A subsequent WHO report published in 2019 provides evidence-based movement behaviour guidelines, encompassing PA, SB, and sleep, for children in their early years [6]. There is a need to be able to measure movement behaviours of pre-school aged children for the purpose of public health monitoring (including in large population samples), to determine compliance with the guidelines, and to assess the effectiveness of interventions and initiatives targeting these behaviours [7].

Proxy report measurement tools (e.g. diary or questionnaire) where a parent/carer reports the movement behaviours of pre-school children are generally agreed to be a more feasible and affordable method compared with other measurement tools, such as device based tools (e.g. accelerometers) [8]. Device based tools are more expensive to use at scale [9] and have additional complexities when measuring multiple movement behaviours, including needing to distinguish between sleeping, SB, and removal of the device [10, 11]. Moreover, there is no consensus on the optimal analysis methods of accelerometer data in this age group [12]. Although there are limitations of proxy reported tools including social desirability and recall bias, they provide important contextual information on the type of activities undertaken and are useful tools for monitoring and surveillance of movement behaviours [13].

To date and to our knowledge, no proxy report measurement tool exists that concurrently captures these separate movement behaviours with the ability to assess adherence to movement behaviour guidelines in this age group [14–16]. A need for further evidence on measurement tools able to assess the movement behaviour of young children, to detect compliance with the guidelines, and to enable comparisons between studies assessing these behaviours, has been reported [6]. Further, most studies examining the quality (validity and reliability) of tools used to measure movement behaviours do not provide information on the development of tools or content validity [15–19], or very minimally describe these properties [e.g., 20, 21].

Content validity is defined as: *‘the degree to which the content of an instrument is an adequate reflection of the construct to be measured’* [22]. Content validity relates to the relevance, comprehensiveness, and comprehensibility of a measurement tool for the construct, target population, and context of use. Content validity is central to the quality of proxy reported tools; researchers suggest that it is initially the most important measurement property to examine, prior to further evaluation of the reliability, validity, and responsiveness of a tool [23]. As content validity is difficult to assess, it is suggested that a thorough development of tool study should be conducted, involving the target population and topic relevant researchers [23, 24]. Content validity is important to ensure minimal discrepancies between the behaviours that pre-school children engage in and what the measurement tools are assessing [23, 25].

Therefore, the aim of this study was to co-design and assess the content validity of a measurement tool ‘Movement Measurement in the Early Years’ (MoveMEY) for the purpose of measuring movement behaviour guidelines of pre-school aged (3–4 years) children.

## Methods

### Overview

The development and content validity assessment of the MoveMEY measurement tool was conducted using the Consensus-based standards for the selection of health measurement instruments (COSMIN) guidelines [23, 25]. COSMIN guidelines have been recommended for studies reporting the development and content validity of measurement tools used to examine PA and SB [23, 26]. The guidelines suggest three main aspects that must be assessed to determine content validity; to ensure that this study meets these criteria, we use the same terminology as suggested by COSMIN throughout this article (See Table 1). The COREQ checklist for reporting qualitative research was also used [27] (additional file 1).

**Table 1:** Constructs and definitions used to determine content validity

*Construct*	*Definition (directly extracted from* [25]*)*
1. Relevance	*All items should be relevant for the construct of interest within a specific population and context of use.*
2. Comprehensiveness	*No key aspects of the construct should be missing.*
3. Comprehensibility	*Items should be understood by participants as intended.*

**Fig. 1:**
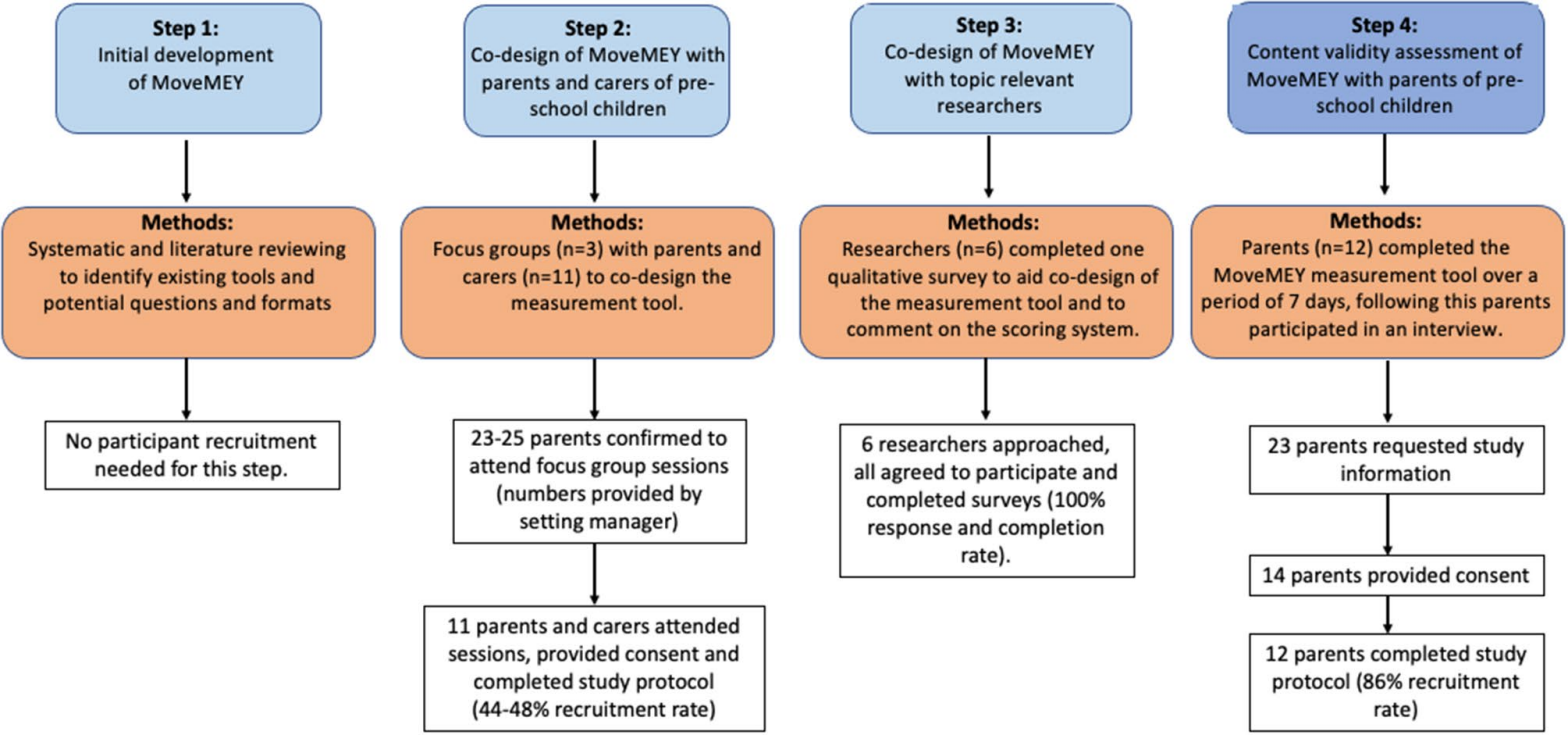
Overview of Methods and Participants

For this study, four steps were followed (see Fig. 1):

1. Tool initially developed based on extensive searching of the literature and mapping questions to global movement behaviour guidelines for pre-school children (6).
2. Development of MoveMEY through focus groups with parents and carers (hereon collectively termed ‘*carers’*) of pre-school children using the initial tool as a stimulus for discussion (**Step 2**: data collection December 2019 and January 2020)
3. Qualitative survey consultation with topic relevant researchers (heron termed ‘*researchers’*), to aid in appropriate development of MoveMEY (**Step 3**: data collection December 2019 and January 2020).
4. Content validity (relevance, comprehensiveness, and comprehensibility) assessed through interviews with carers of pre-school children following piloting of MoveMEY (**Step 4**: data collection August 2021 and January 2022).

Detailed information about (A) Participants, (B) Methods and procedures, and (C) Data analysis, are described in turn below. COSMIN guidelines suggest that sample sizes of ≥ 7 participants are required for an optimal rating in qualitative development of tool studies [23, 25]. However, this was not used as a restriction, and participants were recruited and data collected until sufficient and adequate information and understanding had been reached at each stage of the development of tool process [28].

### Participants

For the focus groups **(Step 2)** and interviews (**Step 4)**, participants were parents and carers of pre-school children (aged 3–4 years old) recruited through early years settings (children’s centres, nurseries, and schools with pre-school provision) in the North of England, United Kingdom (UK). We aimed to target parents and carers living in the most deprived areas in the UK, due to underrepresentation of these groups in measurement literature [16]. As such, we used purposive sampling to select early years settings (hereon termed ‘*settings’*) in the highest quartile of most deprived areas in the UK, obtained by the index multiple deprivation (IMD) score [29]. Settings were approached by telephone, e-mail, or via the personal network of the researchers. For the focus groups (**Step 2**), an opportunity sampling method was used at the participating settings, whereby posters and information sheets were provided to carers of the children describing the purpose and reasons for conducting this research. In one instance, the lead researcher (SMP) attended the setting several times to informally chat with carers about the research and to build rapport with the centre. Children were able to be present during the focus group sessions to promote inclusivity. For the qualitative survey consultation (**Step 3**), researchers were recruited via the personal network of the first and second author of this study and were invited via email. For the content validity study (**Step 4**), all research took place remotely due to the ongoing COVID-19 pandemic. Recruitment was limited to English speaking carers, due to the study materials and diary only being available in English at this time. A different group of carers to those involved in the development of MoveMEY (**Step 2**) were recruited using an opportunity sampling method, with information about the study provided to carers of pre-school children through several methods. This involved:

1. The lead researcher (SMP) contacting settings through telephone and e-mail, using purposive sampling for those in the highest quartile of most deprived areas in the UK, as assessed by the IMD score [30].
2. A local public health practitioner distributing the study poster to nurseries and schools in the area (County Durham).
3. Snowballing recruitment whereby existing participants provided the study recruitment poster to family and friends.

For the content validity study only (**Step 4**), participants received £20 in Love2Shop vouchers upon completion of the study and children received ‘MoveMEY’ stickers.

### Methods and procedures

#### Step 1 – Initial development of MoveMEY

The development process for the initial version of the MoveMEY measurement tool included: (1) a systematic review of measurement tools used to examine PA and SB of pre-school aged children [16]; (2) a literature review of measurement tools used to examine sleep of pre-school aged children, later formalised in the form of a systematic rapid review [15]; (3) conducting a new search to identify and screen the literature of published journal articles examining all three movement behaviours of pre-school aged children with a sample of more than 100 participants, to identify measurement tools used, and (4) screening of current worldwide activity surveillance systems, to identify questions currently being implemented. This step resulted in the initial development of the MoveMEY tool, which was informed by information obtained from the multiple methods of literature screening and by mapping across to the current (2019) WHO movement behaviour guidelines for pre-school aged children [6]. When completing additional literature screening of published journal articles whereby the movement behaviours of pre-school aged children had been examined at scale, we initially searched for research including > 1000 children, as this is what is often seen in surveillance systems as ‘large scale’. However, when scoping the literature, it was apparent that there were minimal studies examining the behaviours collectively in this amount of pre-school aged children (n = 1). Therefore, it was decided that it would be more pragmatic to extend the searches to studies that included more than 100 participants in their final sample.

#### Step 2 – Development of MoveMEY with carers of pre-school aged children

Focus groups (**Step 2**) with carers of pre-school aged children were conducted to co-design the measurement tool, in an available room within the participating setting. The sessions were conducted by the lead researcher (SMP), as part of her PhD research, and who was specifically trained in focus group research. Detailed focus group procedures are previously published [31]. Following informed consent and completion of a demographic information questionnaire, participants were provided with the initial version of MoveMEY (developed in **Step 1**) to act as a stimulus and help facilitate discussion. The purpose of this step was to develop the tool with carers, rather than the tool be developed for them. It was made clear that the measurement tool could be completely re-designed, re-formatted, could include different items, and that items could be added or removed. The topics of the focus group concentrated on the development and design, including on the content and format, of a new tool to assess movement behaviour of pre-school aged children. Discussions centred around identifying what kind of tool would be best, what the tool should look like, as well as discussions around the types, frequency, and duration of activities that their pre-school child engages in to ensure that the tool would appropriately capture the behaviours.

#### Step 3 – Development of MoveMEY with topic relevant researchers

Qualitative surveys (**Step 3**) were completed by researchers using the same initial tool presented to carers in the focus groups (developed in **Step 1**), in parallel to the focus groups **(Step 2)**, to aid development of the tool. Researchers were sent a copy of the initial MoveMEY questionnaire, the scoring sheet, and one free text response qualitative survey. Qualitative survey questions were devised using a range of references and resources [6, 17, 22, 32–34] and included space for any additional comments or suggestions.

#### Step 4 – Content validity assessment with carers of pre-school children

Interviews with parents of pre-school aged children were conducted by the lead researcher (SMP) following piloting of MoveMEY, to assess the content validity (relevance, comprehensiveness, and comprehensibility) of the MoveMEY tool. Following informed consent (by way of a signed and digitally returned consent form), participants completed a demographic information questionnaire and the MoveMEY measurement tool over a period of 7 days. Following completion of MoveMEY, semi-structured telephone interviews were conducted with the participant. The questions on the interview guide were developed based on the quality criteria for good content validity outlined by COSMIN [23, 25]. Participants returned their completed MoveMEY diary back to the lead researcher via post.

For Step 2 and 4 ethical approval was obtained from the Department of Sport and Exercise Sciences ethics committee at University of Durham, UK.

### Data analysis

The focus groups (**Step 2**) and interviews (**Step 4**) were voice recorded and transcribed verbatim. Whilst some field notes were made during both the focus groups and interviews, analysis was primarily based on the transcripts from the voice recordings. The lead researcher (SMP) conducted data analysis. A thematic analysis approach [35, 36] was used in **Step 2, 3** and **4** to code the data to:

- ***Step 2***: Summarise the focus group discussions to create the first official version of MoveMEY.
- ***Step 3***: Summarise the responses to the qualitative surveys, identify overarching themes, and compare coded responses and themes with the responses from carers to adapt MoveMEY.
- ***Step 4***: Summarise the responses from the interviews to determine the content validity of MoveMEY. Based on the results of the interviews, minor amendments were made to MoveMEY.

The final version of MoveMEY was checked by an existing public engagement group including parents of pre-school children, who were recruited through Fuse. Fuse is a Centre for Translational Research in Public Health, based in the North East of England. This public engagement group did not include any participants from Step 2 or Step 4.

Themes for each section (**Step 2, 3**, and **4**) were summarised under the following categories: (1) Format of MoveMEY, (2) Content of MoveMEY, and (3) Scoring of MoveMEY (following the content validity assessment). Any further relevant data that did not fit under these categories were included as additional themes. The completed MoveMEY diaries (from **Step 4**) were scored by the lead researcher to ensure the scoring sheet was appropriate, and the diaries were screened to determine any additional concerns such as if any questions were consistently unanswered that had not been raised through the interviews.

## Results

### Participants

Three focus groups with a total of eleven carers (parents and nursery teachers) of pre-school children took place in early years settings in the North of England, typically lasting between 45 min and 1.5 h (**Step 2**). Children were present in two of the three focus groups, with nursery teachers also present in one of these focus groups to care for the children. Six topic relevant researchers, working in universities in the United Kingdom, participated in the qualitative surveys (**Step 3**). Researchers were in the movement behaviour field, including having expertise in child public health, measurement tool development and evaluation, and physical activity, sedentary behaviour, and sleep of young children. Twelve telephone interviews with parents of pre-school children took place, typically lasting between 25 and 40 min (**Step 4**). A total of 23 parents requested study information, of these, 14 provided consent, and 12 parents (reporting on 13 children) completed the study protocol. Of the two parents who provided consent but did not complete the study, one reported that both of her children were unwell with Covid so could no longer participate, and the other was moving jobs and did not arrange the interview. Children were often present with their parents during the telephone interviews. Figure 1 provides an overview on the participants for each step of the development and content validity assessment of MoveMEY. Table 2 provides information on the demographic characteristics of the participants in **Step 2** and **Step 4** of this research.

**Table 2:** Demographic characteristics of parent and carer participants (Step 2 and 4)

Demographic characteristics		Step 2	Step 4
Number of participants		11	12 reporting on 13 children
Sex of parent (%)	Female	100	92
	Male	0	8
Ethnicity of parent (%)	White British	100	92
	Latvian	0	8
Age of parent/carer (years)	Median	29	37
	Range	21 to 61	27–40
Age of pre-school child (years)	Median	3.7	3.8
	Range	3.3–4.9	3.1–4.11
Sex of child (%)	Female	Question not asked	31
	Male		69
Parental reported ethnicity of child (%)	White British	Question not asked	100
Education level (%)	Bachelor degree or higher	9	92
	A levels or equivalent	9	8
	Diploma in higher education/BTEC or equivalent	18	0
	GCSE’s or equivalent	27	0
	Vocational qualifications	18	0
	No formal qualifications	9	0
	Did not specify	9	0
Employment status (%)	Working full-time	36	34
	Working part-time	27	50
	Looking after the home	9	8
	Not working	27	8
Household income per year (%)	< £4,999	0	8
	£5,000 - £9,999	9	0
	£10,000 -£14,999	9	8
	£15,000 - £19,999	18	8
	£20,000 - £24,999	18	0
	£25,000 - £29,999	18	17
	£30,000 - £34,999	0	0
	£35,000 - £39,999	9	8
	£40,000 - £44,999	0	8
	£45,000 - £49,999	0	8
	£50,000 - £74,999	0	8
	£75,000 - £99,999	0	17
	£100,000 or more	0	0
	Don’t know	18	0
	Prefer not to say	0	8
*Index Multiple Deprivation quintile (%)	1	81	42
(1 = most deprived, 5 = least deprived)	2	9	25
	3	0	17
	4	0	8
	5	9	8

^*^ Step 2 based on 2015 IMD classification [29], Step 4 based on 2019 IMD classification [30].

### Step 1 - Initial development of MoveMEY

The systematic reviews of the literature revealed two promising proxy reported tools for measuring PA and SB of pre-school aged children [21, 37], however, neither of these had a format or content that would provide appropriate output to assess duration of activities or the guidelines. For assessing sleep, only one sleep diary [38] demonstrated reasonable quality and validity, which had some relevant aspects for the initial tool including ‘lights off’ time and morning wake up time to assess sleep duration. The additional searches revealed only one national surveillance system that assessed the movement behaviour guidelines collectively, the Canadian Health Measures Survey, which included accelerometer derived PA and parental reported screen time and sleep [39].

#### Format of MoveMEY

The format of the initially developed tool was based on the Early Years Physical Activity Questionnaire (EY-PAQ) for the PA and SB questions [40], with questions worded as ‘last 7 days’ rather than ‘last month’ due to research suggesting this to be more accurate [33]. The questionnaire was separated into sections to capture the three main behaviours: PA, SB, and sleep. The sleep section of the questionnaire was formatted differently, to be able to capture consistent sleep and wake times.

#### Content of MoveMEY

The content of MoveMEY was developed by the lead researcher (SMP) assuring questions aligned with the WHO movement behaviour guidelines [6], to ensure that the questions appropriately targeted the guidelines. The initial tool, accompanying scoring sheet, and mapping of questions to the guidelines can be found in additional file 2.

#### Scoring of MoveMEY

The scoring of the initial tool was based on the format of the EY-PAQ [40] for the PA and SB questions, as well as the questions on nap time and night wakings. Total sleep duration was determined using the relevant variables (time in bed, time to fall asleep, total minutes awake during the night and total nap duration). Questions on bed time, wake up time, and time to fall asleep were for what is ‘usual’, as such, these applied for every day of the week. ‘Good quality’ sleep was determined based on sleep efficiency, which included ≥ 85% total sleep time from total time in bed to be classified as good sleep quality, based on national sleep foundation recommendations [41]. Wake and bed times were determined to be ‘consistent’ if reported as consistent (classified as within 30 min, based on the definition outlined in [42]) for ≥ 5 days, based on existing questionnaires using this definition [43].

### Step 2 and 3 -Development of MoveMEY with carers of pre-school aged children and topic relevant researchers

#### Format of MoveMEY

Key findings on the format of MoveMEY, from both carers and topic relevant researchers, are displayed in Table 3. Carers consistently reported the importance of the tool capturing differences in routine. This included space to report for home and nursery activity separately, and weekday and weekend activity: ‘*Yeah. That’s a weekday, but then during the weekend it’s-* (P4) ‘*It’s a bit different like us.*’ (P2). Carers suggested that it would be difficult to recall previous week activity of their child, due to the large number and variation of activities. ‘*I think it’s a bit too hard for me this, because like she does so much each day, like all the different things she does through the day, and then you’re having to record them and remember, oh, it’s so confusing, I just don’t understand it’* (P3).

**Table 3:** Development of MoveMEY: key findings on the format from carers and researchers

Carers (n = 11)	Researchers (n = 6)
Daily reporting	Daily reporting
Diary format	Tool may have to be longer to be more meaningful (to be able to collect sufficient and useful data)
Distinguish between weekday/weekend	Distinguish between weekday/weekend
Enable space to report home and nursery activity	Ability to capture home and nursery activity
Report nap time / night wakings daily as they differ from physical activity and sedentary behaviour, in that sleep is more consistent whereas physical activity and sedentary behaviour activities can differ day to day.	Report nap time / night wakings daily as they differ from physical activity and sedentary behaviour, in that sleep is more consistent whereas physical activity and sedentary behaviour activities can differ day to day.
Preferences differed in terms of entering own amount of time for each activity versus ticking boxes	Parents to enter own amount of time for each activity may make MoveMEY less complex
Sleep section slightly different – sleep routines mean that bed/wake time question would not need to be daily	
	Some behaviours may be reported twice – make clear from the start what questions are ahead to avoid this happening

^*Empty cells indicate that the same suggestion had not been made^

Carers and researchers both recommended a diary-based format with daily reporting to aid recall and to capture differences in behaviours between days and routines (e.g., home and nursery). Carers stated this would be much easier and more convenient than retrospective reporting: ‘*I think maybe like this with different days of the week. So you’re not having to sit and work out each day what they’ve done, at least you can have it as like a daily thing*.’ (P5). Researchers stated that due to the complexity of measuring movement behaviour of pre-school children through a proxy report, there may need to be a trade-off between brevity of a tool and the number/type of responses, including that a longer tool may be more meaningful for parents to complete as this may more accurately represent their child’s behaviour and is more likely to provide sufficient and useful data for researchers. Carers noted that daily reporting for different activities would create ‘*more paperwork’* but that ‘*in the long run it’ll be easier’*.

The structure for reporting sleep was regarded slightly differently. Carers suggested that stating ‘usual’ bed and wake times would work due to having strict and consistent bedtimes for their pre-schoolers, but felt it was important to keep space to report differences and reasons for the difference. Similarly, researchers stated that it was important to distinguish between time in bed and time asleep, and to ensure space for parents to supplement their responses to the question on consistency of bed and wake times to state why times may differ. For nap time and night wakings, daily reporting was suggested by both carers and researchers to give a more accurate estimate, as these behaviours can vary substantially depending on the day and different circumstances.

#### Content of MoveMEY

The content of the initial MoveMEY tool was generally well received. Key findings on the content of MoveMEY, from both carers and topic relevant researchers, are displayed in Table 4. Carers stated that the activities outlined were relevant ‘*captured most of what they do*.’ (P11) and comprehensive in terms of the activities that children engage in *‘…I think you’ve covered the basic ones.*’ (P2).

**Table 4:** Development of MoveMEY: key findings on the content from carers and researchers

Carers (n = 11)	Researchers (n = 6)
Initial tool was relevant and covered most of the ‘basic’ activities that children of this age engage in.	Initial tool thought to be relevant, provided an adequate reflection of the movement behaviours of this age group, and appropriately targeted the guidelines.
Locations of physical activity /active play, rather than specific activities.	Locations of physical activity /active play, rather than specific activities.
Distinguish between different types of seated travel (e.g., separate spaces for travelling in car, on the bus).	Separate seated travel question to distinguish between different types of seated travel and to appropriately capture guidelines.
Space to add some additional activities.	Additional activities to be added: ‘scooter’ (for leisure and active travel), soft play and puzzles. ‘Bath time’, ‘sitting whilst eating’ and ‘sitting whilst on the potty’ should be added, to ensure that these seated behaviours were captured.
Term ‘sweating’ not a word parents would use in relation to their pre-school child.	‘Out of breath’ or ‘breathe harder’ may be more relevant alternatives to the term ‘sweat’ for this age group.
Space to report ‘general information’ such as illness, sleep problems or disabilities.	Space to report ‘general information’ such as illness and sleep problems.
Children sometimes use screens before bed so unsure on sleep time.	Screen time before bed may be important additional factor to capture.
Question on bed/wake time consistency- space to explain why differences may be present on certain days.	Question on bed/wake time consistency - space to explain why differences may be present on certain days.
Allow space for active travel versus walking/cycling for leisure.	
Space for ‘additional comments’ to help put the responses into context.	
	Specific activities for sedentary behaviour and screen time
	Report ‘0’ if activity didn’t happen
	Add lay definitions of the behaviours to be captured

^*Empty cells indicate that the same suggestion had not been made^

Similarly, researchers felt that MoveMEY contained relevant questions, represented an adequate reflection of each of the movement behaviours of pre-schoolers, and appropriately targeted the guidelines. It was suggested by both carers and researchers that in some instances stating location of activity may help with recall, such as the question on ‘*outdoor activities’* including options such as ‘*in the park’*, rather than specific activities (e.g. time spent climbing). Carers suggested including example activities to help understand the question, alongside some blank spaces to add any additional activities. ’*So we could just have examples so people don’t think hmm, what have they done?…*’ *Yeah, but then have the choice to also write your own if you can do that.’* (P4). Carers also recommended having ‘*additional comments’* sections to explain responses and provide detail (e.g. provide context around sleeping arrangements, such as child changing bed during the night).

Carers suggested that it may be important to distinguish between the different types of seated travel, as not all were relevant to them. ‘*…So we’re in the car quite a lot when we go out because my partner drives so we go everywhere… So I don’t know if being in a car or on public transport would maybe be relevant*.’ (P9). Likewise, researchers suggested that the question on seated travel should either explicitly state ‘restrained’ or be separated by the different modes of sedentary behaviour so that distinctions could be made between seated activity and restrained activity (e.g., seated whilst on the bus, seated in the car). Carers reported that they do not always know when their child falls asleep, as they may watch TV or play on a tablet before sleeping; highlighting that screen time before bed may be an important factor to include in MoveMEY. Researchers similarly suggested that it may be beneficial to capture screen time before bed. Although not a current guideline, there is accumulating evidence in this area [44], including being recommended by the UK guideline expert committee [45]. This also highlights that sleep latency may be difficult to assess in this age group and, therefore, emphasises the need for clarity in a tool to distinguish between bed and sleep time.

The terminology of ‘sweating’ was not appropriate for young children: ‘*I wouldn’t ask them if she was sweating…*.’ (P5). Researchers suggested that ‘*out of breath’* or ‘*breathe harder’* may be more relevant terminology.

Space to report ‘*general information’* about the pre-school child was recommended, to help put behaviours into context. This included reporting if their child:

1. Is suffering from illness at the time of measurement: *‘…there should be a box here if they were ill, because [child] she never naps now, but if she’s ill she would probably sleep nearly all day’* (P10).
2. Has ‘*sleep problems’*, including night disturbances such as night terrors or to report the ‘*type of sleeper’* that their child is. This question explicitly stated ‘medical’ sleep problems with space to report information about the sleep problem, due to night-time awakenings being common in pre-school children and lack of consensus on what is considered an excessive sleep problem versus normal sleeping behaviours.
3. Has a disability that may impact on movement behaviours.

Researchers suggested that MoveMEY was comprehensible, but for clarity lay definitions should be added to outline the key behaviours. Finally, carers suggested that it may be beneficial to be able to input the amount of time for each activity, as prescribed and specified times may add to the complexity of completing MoveMEY. A concern with this approach was researcher burden of handling the data. The revised MoveMEY measurement tool following this development process outlined in **Step 2 and 3** and accompanying scoring sheet, which was then assessed for content validity in the next step, can be found in additional file 3.

### Step 4 – Content validity assessment with carers of pre-school children.

Interviews with carers found no major concerns with MoveMEY that had been revised following **Step 2** and **Step 3**. MoveMEY was stated to contain relevant items, was comprehensive to detect the range of children’s movement behaviours, and there were no concerns with comprehensibility of MoveMEY and instructions. Despite this, the content validity study allowed further improvements of MoveMEY in line with parental feedback, which we describe below. An overview of the amendments made to MoveMEY following the content validity study can be found in Table 5.

**Table 5:** Overview of the amendments made to MoveMEY following the content validity assessment

Original question in MoveMEY	Concerns	Revised question
**Part one: General Questions**		
1. Does your child have any physical, neurodevelopmental, or medical condition or disability that affects their ability to play and be physically active?	No concerns	No amendments made
2. Does your child have any medical sleep problems, such as night terrors?	No concerns	No amendments made
3. Is your child currently suffering from an illness that may affect their normal behaviours, including being active, movement, sitting or sleep?	Question appearing as though it is only aimed at long term illness, rather than just being unwell during the week of measurement	Changed to include more example so that even short-term illnesses are captured. *‘Is your child currently suffering from an illness, unwell or poorly (short or long term) that may affect their normal behaviours, including being active, movement, sitting or sleep?’*
**Part Two: Physical Activity**		
4a. Please state how many hours and minutes your child spends actively playing **outdoors** in each of the following (activities may include: running around, jumping on a trampoline, climbing, skipping, throw/catch).	Columns for home and nursery activity, but specific row was also available for ‘indoors at nursery’.	Crossed out ‘home’ boxes for the ‘outdoor play at nursery’ row
4b. Did any of these activities make your child ‘huff and puff’ or breathe harder? (Please circle)	Parents did not always feel their child would be out of breath or ‘huffing and puffing’, but that a range of activities would suggest this intensity of activity.	Added more examples of higher intensity activity, including makes child breathless, hot and sweaty, or need a drink or rest
4c. If yes, please state how many hours/minutes of this activity made your child ‘huff and puff’ or breathe harder.		
5a. Please state how many hours and minutes your child spends **actively travelling**, which could include travelling for leisure (e.g. to/from school, the shops, the park) each day.	No concerns	No amendments made
5b. Did any of these activities make your child ‘huff and puff’ or breathe harder? (Please circle)	Parents did not always feel their child would be out of breath or ‘huffing and puffing’, but that a range of activities would suggest this intensity of activity.	Added more examples of higher intensity activity, including makes child breathless, hot and sweaty, or need a drink or rest
5c. If yes, please state how many hours/minutes of this activity made your child ‘huff and puff’ or breathe harder.		
6a. Please state how many hours and minutes your child spends actively playing **indoors** (activities may include: dancing, running around, rough and tumble play, sit and ride push toys).	Columns for home and nursery activity, but specific rows were also available for ‘indoors at home’ and ‘indoors at nursery’.	Removed columns for home/nursery, as there was a row for each of these locations.
6b. Did any of these activities make your child ‘huff and puff’ or breathe harder? (Please circle)	Parents did not always feel their child would be out of breath or ‘huffing and puffing’, but that a range of activities would suggest this intensity of activity.	Added more examples of higher intensity activity, including makes child breathless, hot and sweaty, or need a drink or rest
6c. If yes, please state how many hours/minutes of this activity made your child ‘huff and puff’ or breathe harder.		
Additional comments on physical activity	No concerns	No amendments made
**Part Three: Sedentary Behaviour**		
7. Please state how long your child spends in **screen based** activities whilst in a sitting, reclining or lying position.	No concerns	No amendments made
8. Please state at what time your child last uses a screen before going to bed (e.g. if child watches a film before bed).	Screen time before bed, some children do not watch screens after morning, but parents still found this self-explanatory.	No amendments made
9. Please state how long your child spends **playing and in other activities** whilst sitting, reclining or lying, including **quiet or carpet time**.	No concerns	No amendments made
10. Please state how long your child spends **seated whilst travelling**.	No concerns	No amendments made
Additional comments on sedentary behaviour	No concerns	No amendments made
**Part Four: Sleep**		
11. Please write your child’s usual bed time and wake up time.	Some parents stated variation in bed, sleep, wake and out of bed time – daily reporting of these factors may increase accuracy of tool.	Changed to daily reporting of bed time, sleep time, wake time, out of bed time.
12. On which days of the week is this the case? (Please circle and provide a reason in the box if different)	Removed question due to change in format for sleep questions, meaning that consistency can be detected by the MoveMEY tool without this additional question.	
13. From the time that your child goes to bed, how long does it take them to fall asleep?	Some parents stated variation in bed, sleep, wake and out of bed time – daily reporting of these factors may increase accuracy of tool.	Changed to daily reporting of bed time, sleep time, wake time, out of bed time.
14. From the time that your child wakes up, how long does it take them to get out of bed?	Some parents stated variation in bed, sleep, wake and out of bed time – daily reporting of these factors may increase accuracy of tool.	Changed to daily reporting of bed time, sleep time, wake time, out of bed time.
15. Please state how many times and for how long each time, that your child wakes up during their night-time sleep.	No concerns	No amendments made
16. Please state how many times and for how long each time that your child naps during the day.	No concerns	No amendments made
Additional comments on sleep	No concerns	No amendments made

#### Format of MoveMEY

Parents stated that reporting activity every day was beneficial: *‘But certainly, I think I needed to do it every day ‘cause otherwise I would forget what we’d done’* (P14), that reporting hours and minutes was the best way to recall PA and SB activity, and that the weekly overview helped to show the reality of their children’s movement: *‘…Erm… so, if you just picked like two or three days potentially it would have given you more of a skewed reality. Whereas the week … it gives more of a chance to show reality…’* (P23). The sequence of questions was well received by parents: ‘*I think they were fairly erm… they flowed pretty well to be honest. Erm… so… no, it made a lot of sense that the order that you had them in made a lot of sense’* (P12).

Improvements to MoveMEY included changing the sleep section so that all questions had daily response options, due to variation in the bed and sleep time of the children: ‘*I mean normally there can be a bit of fluctuation some days… so, yeah, I think that potentially given the option to say each time might have been like more reliable’*. (P23). Further, it was suggested that some boxes were irrelevant and could be removed, for example, crossing out ‘*home’* boxes when the question was specific to ‘*outdoor play at school’*. An example of how to complete MoveMEY to improve the clarity on what is expected was added based on a parental suggestion.

#### Content of MoveMEY

MoveMEY was comprehensible, with parents consistently reporting that MoveMEY was ‘*easy to understand’*, ‘*straightforward’*, and ‘*self-explanatory’* and that MoveMEY was ‘*well worded’*, ‘*well structured’*, and ‘*well designed’*. Parents felt that MoveMEY was comprehensive and relevant, that it covered all ‘*age-appropriate activities’* that their children would engage in: ‘*Erm… I think it… everything that a child her age does is covered and more to be honest*…’(P14). Some parents appreciated having space to include additional activities, such as ‘*friend’s party with bouncy castle’* and *‘going to the cinema’*. Others did not feel it was necessary as they felt everything had been covered: ‘*…I didn’t put anything in, in sort of the ‘Other Activity’ just because everything else had… had covered-*’ (P17). It was acknowledged that ‘other’ behaviours may be missed but would potentially be difficult to capture in any tool: ’*general kind of toddling about… you know, just little things like that obviously you can’t really cover that*.’ (P22). However, this was represented as a limitation of proxy reported tools.

Improvements to the content of MoveMEY included altering the wording of the question targeting higher intensity PA. Multiple alternative suggestions for the wording of this question were proposed and are included in the revised format. Further, during the period of measurement, some participating children were suffering from illness that impacted on their usual behaviours. One parent interpreted the question to mean long-term illness ‘*I just presumed like an illness, you know, like a long-term illness for some reason… I didn’t think that, you know, him being poorly for the short spell.*’ (P18). As such, this question was amended to ensure that short-term illness for the period of measurement would also be captured.

#### Usability of MoveMEY

Parents suggested that MoveMEY was easy to complete daily: ‘*But yeah, I think it’s all right, it’s not a massive task, it’s just a couple of minutes, a few minutes a day, you know, just to write a quick diary entry, isn’t it?*’ (P18). Parents stated that when children were in the care of other family members, they would ask the family member to keep a log of the child’s activity and/or would obtain the information verbally when collecting their child. When children were at nursery, parents used strategies including asking nursery if they could complete the diary during the day or report to parents at the end of the day, extracting information uploaded to social media by the nursery, or through information posted on the phone applications (Apps) used by the nurseries.

#### Scoring of MoveMEY

In some instances, the overall reported time exceeded the 24 h of the day. There are several reasons why this may be: (1) Sleep questions as part of the tested tool were broad asking for ‘usual’ sleep rather than daily reported. However, the main amendment to MoveMEY includes that sleep is reported daily. (2) Parents overestimating the amount of time their children engage in PA, and in some instances SB too, which is a known challenge of proxy reported tools. (3) Cross-over of activity; parents reported that their children sometimes engage in two behaviours simultaneously (e.g. walking whilst playing on a tablet). Therefore, the same time-period was recorded twice. The diaries took on average around 10 min to score manually.

### Description of the MoveMEY measurement tool

**Fig. 2:**
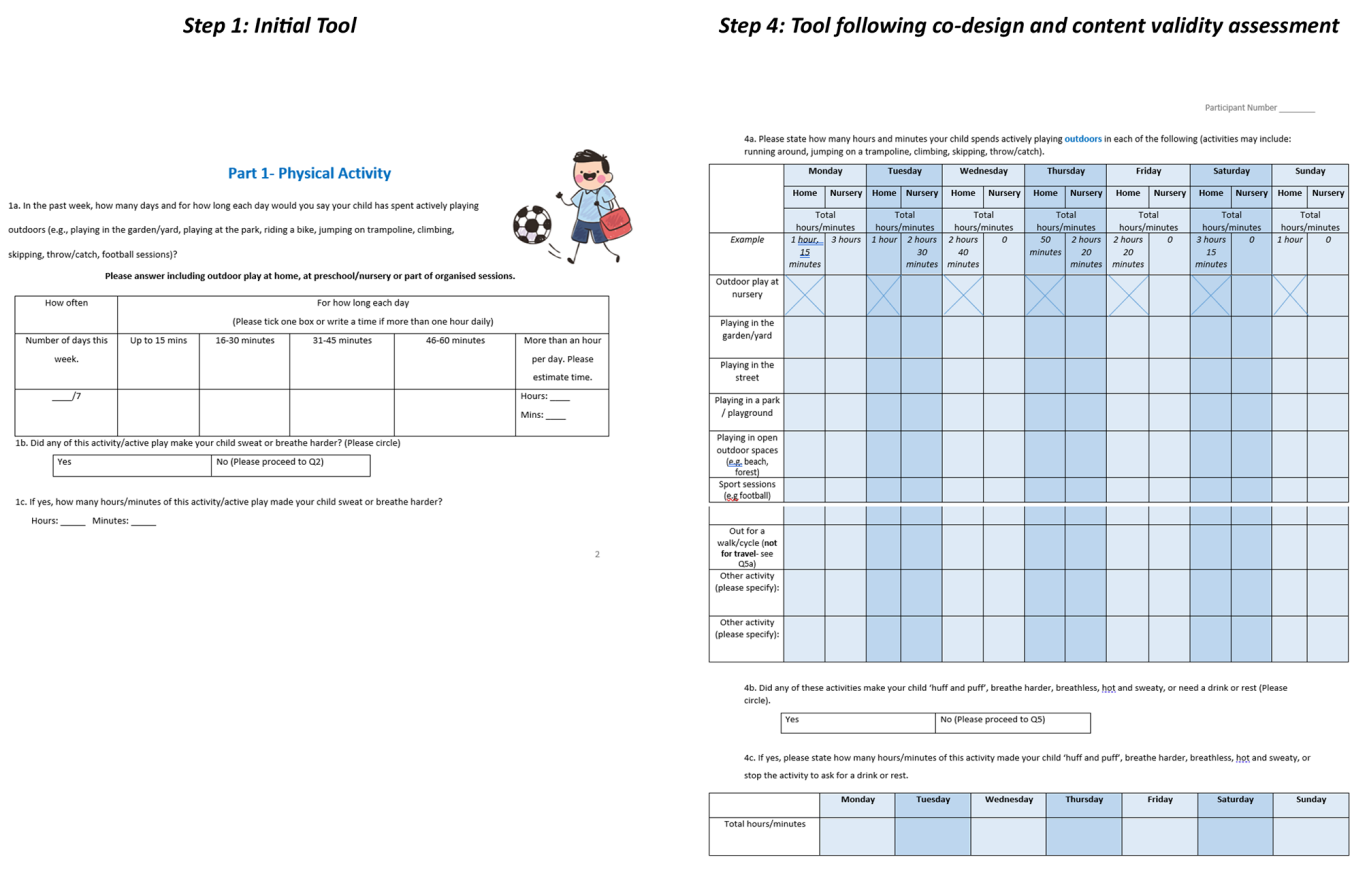
Illustration of initial tool (Step 1) to finalised tool (Step 4)

The final developed version of MoveMEY consists of four sections: (1) General questions: (2) Physical activity, (3) Sedentary behaviour and (4) Sleep. In general questions, carers can report information on whether their child has any disabilities, sleep problems, or illnesses. In section two, carers can record duration of time their child spends in different outdoor and indoor physical activities, and active travel. There is space to record how many hours and minutes of this activity is of higher intensity. In section three, carers can report duration of time their child engages in different sedentary screen-based activities, seated whilst travelling, and engaging in different sedentary pursuits (e.g. playing with toys, crafts). One question asks the last time the child uses a screen before bed. In section four, parents report what time their child goes to bed, to sleep, wakes up, and gets out of bed. Alongside how many times and how long each time their child wakes during the night and naps during the day to determine sleep duration, quality, and consistency of their child’s sleep. A scoring sheet accompanies MoveMEY that can be used to determine whether children meet the thresholds for the guidelines. The final developed version of MoveMEY, ready for further evaluation, along with accompanying scoring sheet, and how questions map to the guidelines can be found in additional file 4. Figure 2 provides an illustration on how the tool changed from the initial version (Step 1) to the finalised version following co-design and content validity assessment (Step 4).

## Discussion

This study outlines the rigorous development and content validity of Movement Measurement in the Early Years (MoveMEY), a newly developed paper based seven-day daily reported diary that measures the behaviours of PA (frequency, duration, intensity, and type), SB (frequency, duration, and type) and sleep (duration and quality) of pre-school children (aged 3 or 4 years old). MoveMEY was co-designed with parents and carers of pre-school children, as well as topic relevant researchers. MoveMEY can be administered for use by carers of pre-school aged children without the need for researcher assistance. The intended application of MoveMEY is to determine adherence to the WHO movement behaviour guidelines [6].

An initial tool was developed and subsequently co-designed with carers (parents and nursery teachers) and researchers, including substantial modifications to the design, format, and content of MoveMEY. Main findings from the co-design process included a change from a short questionnaire to a detailed 7-day diary based format, separated by the distinct constructs (PA, SB and sleep), and including general information questions on child’s health status. These changes were suggested by the carers included in the development process; with the granularity of the diary format allowing for a more detailed overview of children’s movement behaviours to aid in being able to detect the behaviours children engage in. This format was suggested for various reasons including daily reporting being easier to recall (and thus providing more accurate and meaningful responses), providing the ability to distinguish between weekday and weekend activity, and ensuring that there was not an over/underestimation of certain behaviours such as naps and night wakings that do not always occur consistently throughout the week. Throughout the stages of development carers consistently stated the variability between days in the activity of their young children, which highlighted the importance of a tool that can capture this variation and intricacies between days to provide a more accurate representation of pre-school children’s movement behaviour. Further to this, MoveMEY was split so there was space to report PA and SB separately for both home and nursery. This was recommended to improve the likelihood of detecting nursery-based activity, as nursery teachers could help with completing the tool.

During the development of MoveMEY, carers and researchers found that the initial version included relevant activities for children of this age, and researchers felt the items of MoveMEY appropriately captured the content of the guidelines. Multiple suggestions were made to make MoveMEY more comprehensive and easier to complete, which were relatively consistent between carers and researchers. For example, separating the different modes of seated travel and changes and clarification on terminology. Some substantial additions included adding a space for ‘additional comments’ at the end of each section, a question on screen use before bed, and a section on ‘general information’ to report disabilities, sleep problems and illness.

The content validity study confirmed that MoveMEY is comprehensive and relevant to pre-school children’s movement behaviours, was not missing relevant items, was comprehensible and easy to use. Some amendments were made to MoveMEY following the content validity study, in line with parental feedback, including adding more examples to questions aiming to detect moderate to vigorous PA and crossing out/removing some irrelevant columns (e.g. crossing out ‘nursery’ boxes when the question was specific to ‘home’). The most substantial amendment was the transition from questions asking about ‘usual’ bed, sleep, wake, and out of bed time, to daily reporting, as per the rest of the diary. This resulted in removal of a question on consistency of bed/wake time, as this was no longer necessary. The revised and finalised version of MoveMEY was then checked by a public engagement group of parents, who confirmed that the final version was appropriate and did not raise any further concerns.

MoveMEY is a novel tool with distinct differences compared to existing parental reported tools examining movement behaviour. To our knowledge, there are currently no available tools, that have undergone measurement property evaluation that would be suitable for determining the movement behaviour guidelines of pre-school children [14–16]. Existing parental reported tools examining movement behaviours of young children do not have the ability to determine the prevalence of meeting the guidelines due to questions asked and response options not aligning with the movement behaviour guidelines [46–48]. MoveMEY therefore presents the first co-designed tool undergoing measurement property evaluation that would have the ability to determine the movement behaviour guidelines of pre-school children.

Although at first intended to be a brief questionnaire, through the co-design process with parents and carers, the developed tool is a seven-day diary, separated by the distinct constructs (physical activity, sedentary behaviour, and sleep). MoveMEY is completed daily for a period of seven days by the child’s parent or primary caregiver, with additional information provided from nursery teachers and other caregivers, such as grandparents, to capture movement behaviour when children are being cared for by others. The granularity of the diary format allows for a more detailed overview of child’s movement behaviours including frequency, duration, and type of activities. Whilst an advantage of this tool is the granularity in which it can detect movement behaviours, which may be beneficial in providing more accurate and detailed results than previous parental reported tools, it is plausible that this may be quite intensive for large-scale measurement. However, there is evidence of success using the ‘time use survey’ (> 4500 participants in the published studies after data cleaning), whereby participants self-report activity they engage in every 10 min for two 24-hour periods [49, 50]. In addition, given that the present tool was co-designed by parents and carers, with the majority reporting that they did not find completing the tool time consuming, there can be confidence that this is the type of tool preferred by parents and carers of pre-schoolers. However, it is necessary for future research examining the tool to be tested with larger samples to determine the applicability of the tool for large scale measurement.

### Strengths and limitations

The main strength of this study is the in-depth development process and assessment of content validity, using the recommended COSMIN guidelines for a high quality development of tool study [26]. Involvement of parents/carers and researchers in both the development and content validity assessment of the new measurement tool was advantageous, as parents and carers are the experts of their children’s behaviour, and are best suited to state the activities their children engage in. Although this tool was developed through a rigorous development process, including the first important step of ensuring content validity, which included key insights from parents and carers, there are some sources of error and bias with parental reported tools that may be unavoidable. This includes that parents are not always with their children and may have to estimate time due to the sporadic and intermittent nature of children’s behaviours or any night waking. Whilst these limitations must be acknowledged and may limit the accuracy of parental reported tools for such young children, there is still a necessity for tools of this kind [13], and the detailed information the tool can offer in terms of the type and duration of activity across different days of the week is valuable. In addition, an accepted challenge of measuring sleep of young children by proxy report is that this may be difficult to accurately assess if the parent is asleep themselves and/or in a separate room. Despite this, proxy report is still the most widely used method for assessing sleep, and to date, there is limited research examining alternative methods for assessing habitual sleep in this age group [15].

A further strength is that the recruitment strategy deliberately targeted lower socioeconomic status (SES) groups, whose views are often underrepresented in measurement literature [15, 16]. Participants in both the development and content validity assessment of MoveMEY largely consisted of individuals living in the most deprived regions in the UK, based on the IMD [29, 30]. However, participants in the content validity study (step 4) were highly educated (92% educated to Bachelor degree level or higher). Using the Flesch-Kincaid readability calculator [51], most items of the final version of MoveMEY were judged as ‘easy to read’ and suitable for those aged 10–11 years, with some items scored as ‘Plain English’ and being suitable for those aged 13–14 years. Despite this resulting sample, a targeted recruitment approach was an important consideration, as even when using this targeted approach, we found that a large proportion of participants were highly educated and had high incomes, as such, it was particularly pertinent to aim to capture some representation from parents and carers from lower SES backgrounds. Although it is unknown if the development of MoveMEY would have differed if we had not used this targeted recruitment approach, and rather had a more broader recruitment strategy, it was important to focus recruitment on lower SES groups for several further reasons, including: (1) Inequalities in representation in measurement literature, with limited evidence for individuals from lower SES backgrounds [15, 16] and (2) Due to persistent inequalities in health and health outcomes, interventions and initiatives aimed at improving movement (and related e.g. eating) behaviours of young children are often targeted at lower SES groups [e.g. 52, 53] and so it is particularly important that tools are relevant, usable, and understandable to these groups. Successfully recruiting low SES families for this type of research project is an accepted challenge [54]. A plausible reason that the recruitment strategy focusing on lower SES groups may have been less successful in Step 4 is due to this part of the research taking place during the Covid-19 pandemic, which resulted in heightened responsibilities for early years settings which restricted the degree to which settings could engage in research (e.g. needing to limit unnecessary contact) and staff shortages including increased staff absences due to Covid. This also limited the extent to which recruitment and data collection could take place face to face, which may have impacted the diversity of the sample. Stuber and colleagues [54] suggest visiting the location of the target group and involving key community members as strategies to help achieve success with recruitment of low SES groups in research. We were unable to employ such methods due to the specific circumstances under which this research took place (during the covid-19 pandemic).

A limitation of the work is that despite trying to achieve a diverse sample by recruiting settings in one of the most ethnically diverse cities in the UK [55], the sample in the present study was not ethnically diverse, as such, generalisability of the findings across different ethnic groups is unknown. Further evaluation of MoveMEY should include ethnically diverse samples. The ways in which this may be achieved would include several strategies: (1) Trying to access a gatekeeper with whom individuals from different ethnic backgrounds may feel more able to identify with, (2) Public engagement and involvement with individuals of different ethnic origin, to identify if the recruitment materials (e.g. recruitment posters) can be made more inclusive, (3) Aiming for face to face recruitment in settings in ethnically diverse communities. MoveMEY has been developed in English, with only fluent English speakers involved in the development and content validity assessment. This limited the inclusivity of our research, and the applicability and suitability of MoveMEY to families in other countries would require further research.

In addition, the use of a qualitative survey with a small number of topic relevant researchers may have limited the scope of responses in comparison with other possible methods, such as a Delphi study. However, the purpose of this stage was to ensure that an overview of perspectives from topic relevant researchers with extensive experience in the field of child public health, measurement of physical activity and related behaviours, and/or knowledge on movement behaviour guidelines, were involved with this process, to ensure the tool was appropriate for research in this area.

The age group of children aged 3–4 years was chosen to ensure consistency between the target population of the tool and the guidelines in this age group [6]. However, this may limit the scope of the tool to this age group only.

### Implications of findings and future research

This study highlighted the importance and value of rigorous tool development with the target population, in ensuring that the tool is relevant, comprehensive, comprehensible, and in a suitable format for end users. A key implication of this work is the methodological advancement that it provides. To date, no measurement tools for assessing movement behaviours of pre-school children have been developed and assessed for content validity with both parents/carers of pre-schoolers and topic relevant researchers [14]. In line with recent works in older age groups (9–12 year old children) [56], this study serves as a model to help inform procedures for future development of measurement tools used to assess movement behaviour.

This is a novel piece of work as, to our knowledge, this presents the first study that has developed a single tool that could be used to assess compliance with the recent WHO movement behaviour guidelines for pre-school children [6]. MoveMEY, the newly developed tool presented here, demonstrates good content validity including having the ability to detect ‘type’ of activity such as screen time. There are some specific areas for future research to conclude on the quality and accuracy of MoveMEY, including examining the:

- Convergent validity of MoveMEY by evaluating MoveMEY alongside a range of reference methods, such as direct observation, accelerometer, and sleep diaries to determine if MoveMEY provides similar outcomes to valid pre-existing methods, including research into the minimum number of days needed for a valid week.
- Test-retest reliability of MoveMEY, alongside a comparison tool to determine if MoveMEY is stable at detecting the behaviours, and to determine whether any differences reported are because of changes attributable to the tool versus changes in behaviour.
- Feasibility of MoveMEY at scale, through evaluation with large samples of children, particularly with sub-groups not involved in the development of the tool.
- Variation in tool administration, including online (app) versus paper-based tools. This would apply to MoveMEY, but also as an area of research more generally to examine the effectiveness and accuracy of tools in these different formats.

## Conclusion

The rigorous development and content validity assessment of MoveMEY, with parents and carers of pre-school aged children and topic relevant researchers resulted in a relevant, comprehensive, and comprehensible 7-day daily reported diary that can be used to measure movement behaviour (physical activity, sedentary behaviour, and sleep) of pre-school children (aged 3–4 years). Assessment of the convergent validity (in comparison with reference methods of direct observation, accelerometer, and sleep diary) and test-retest reliability of MoveMEY is an important next step to conclude the accuracy of the measurement tool.

### Electronic supplementary material

Below is the link to the electronic supplementary material.

Additional file 1: COREQ Checklist

Additional file 2: Initial MoveMEY tool

Additional file 3: Co-designed MoveMEY tool assessed for content validity

Additional file 4: Final developed version of MoveMEY

## Data Availability

Data supporting the conclusions of this article are included within the article and additional files.

## List of abbreviations

COSMIN: Consensus-based Standards for the selection of health Measurement Instruments
MoveMEY: Movement Measurement in the Early Years
WHO: World Health Organization

## References

[1] Chaput JP, Gray CE, Poitras VJ, Carson V, Gruber R, Birken CS (2017). Systematic review of the relationships between sleep duration and health indicators in the early years (0–4 years). BMC Public Health.

[2] Carson V, Lee EY, Hewitt L, Jennings C, Hunter S, Kuzik N (2017). Systematic review of the relationships between physical activity and health indicators in the early years (0–4 years). BMC Public Health.

[3] Carson V, Kuzik N, Hunter S, Wiebe SA, Spence JC, Friedman A (2015). Systematic review of sedentary behavior and cognitive development in early childhood. Prev Med.

[4] Rosenberger ME, Fulton JE, Buman MP, Troiano RP, Grandner MA, Buchner DM (2019). The 24-Hour activity cycle: a new paradigm for physical activity. Med Sci Sports Exerc.

[5] World Health Organization. Report of the Commission on Ending Childhood Obesity: implementation plan: executive summary. 2017. Available at: WHO-NMH-PND-ECHO-17.1-eng.pdf.

[6] World Health Organization. Guidelines on physical activity, sedentary behaviour and sleep for children under 5 years of age. 2019. Available at: Guidelines on physical activity, sedentary behaviour and sleep for children under 5 years of age (who.int). 31091057

[7] Aubert S, Brazo-Sayavera J, González SA, Janssen I, Manyanga T, Oyeyemi AL (2021). Global prevalence of physical activity for children and adolescents; inconsistencies, research gaps, and recommendations: a narrative review. Int J Behav Nutr Phys Activity.

[8] Brusseau T, Fairclough S, Lubans D. The routledge handbook of youth physical activity. Routledge; 2020.

[9] Pedišić Ž, Bauman A (2015). Accelerometer-based measures in physical activity surveillance: current practices and issues. Br J Sports Med.

[10] Janssen X, Cliff DP (2015). Issues related to measuring and interpreting objectively measured sedentary behavior data. Meas Phys Educ Exerc Sci.

[11] Quante M, Kaplan ER, Rueschman M, Cailler M, Buxton OM, Redline S (2015). Practical considerations in using accelerometers to assess physical activity, sedentary behavior, and sleep. Sleep Health.

[12] Lettink A, Altenburg TM, Arts J, Van Hees VT, Chinapaw MJM (2022). Systematic review of accelerometer-based methods for 24-h physical behavior assessment in young children (0–5 years old). Int J Behav Nutr Phys Activity.

[13] Sattler MC, Ainsworth BE, Andersen LB, Foster C, Hagströmer M, Jaunig J (2021). Physical activity self-reports: past or future?. Br J Sports Med.

[14] Arts J, Gubbels JS, Verhoeff AP, Chinapaw MJM, Lettink A, Altenburg TM (2022). A systematic review of proxy-report questionnaires assessing physical activity, sedentary behavior and/or sleep in young children (aged 0–5 years). Int J Behav Nutr Phys Activity.

[15] Phillips SM, Summerbell C, Ball HL, Hesketh KR, Saxena S, Hillier-Brown FC (2021). The validity, reliability, and feasibility of Measurement Tools used to assess sleep of pre-school aged children: a systematic Rapid Review. Front Pediatr.

[16] Phillips SM, Summerbell C, Hobbs M, Hesketh KR, Saxena S, Muir C (2021). A systematic review of the validity, reliability, and feasibility of measurement tools used to assess the physical activity and sedentary behaviour of pre-school aged children. Int J Behav Nutr Phys Activity.

[17] Kelly P, Fitzsimons C, Baker G (2016). Should we reframe how we think about physical activity and sedentary behaviour measurement? Validity and reliability reconsidered. Int J Behav Nutr Phys Activity.

[18] Hidding LM, Altenburg TM, Mokkink LB, Terwee CB, Chinapaw MJM (2017). Systematic review of Childhood Sedentary Behavior Questionnaires: what do we know and what is next?. Sports Med.

[19] Hidding LM, Chinapaw MJM, van Poppel MNM, Mokkink LB, Altenburg TM (2018). An updated systematic review of Childhood Physical Activity Questionnaires. Sports Med.

[20] Bacardi-Gascón M, Reveles-Rojas C, Woodward-Lopez G, Crawford P, Jiménez-Cruz A (2012). Assessing the validity of a physical activity questionnaire developed for parents of preschool children in Mexico. J Health Popul Nutr.

[21] Dwyer GM, Hardy LL, Peat JK, Baur LA (2011). The validity and reliability of a home environment preschool-age physical activity questionnaire (Pre-PAQ). Int J Behav Nutr Phys Activity.

[22] Mokkink LB, Terwee CB, Patrick DL, Alonso J, Stratford PW, Knol DL (2010). The COSMIN study reached international consensus on taxonomy, terminology, and definitions of measurement properties for health-related patient-reported outcomes. J Clin Epidemiol.

[23] Terwee CB, Prinsen CAC, Chiarotto A, Westerman MJ, Patrick DL, Alonso J (2018). COSMIN methodology for evaluating the content validity of patient-reported outcome measures: a Delphi study. Qual Life Res.

[24] Olshansky E, Lakes KD, Vaughan J, Gravem D, Rich JK, David M (2012). Enhancing the Construct and Content Validity of Rating Scales for Clinical Research: using qualitative methods to develop a rating scale to assess parental perceptions of their role in promoting infant Exercise. Int J Educ Psychol Assess.

[25] Terwee CB, Prinsen CA, Chiarotto A, De Vet H, Bouter LM, Alonso J (2018). COSMIN methodology for assessing the content validity of PROMs–user manual.

[26] Degroote L, DeSmet A, De Bourdeaudhuij I, Van Dyck D, Crombez G (2020). Content validity and methodological considerations in ecological momentary assessment studies on physical activity and sedentary behaviour: a systematic review. Int J Behav Nutr Phys Act.

[27] Tong A, Sainsbury P, Craig J (2007). Consolidated criteria for reporting qualitative research (COREQ): a 32-item checklist for interviews and focus groups. Int J Qual Health Care.

[28] Braun V, Clarke V (2021). To saturate or not to saturate? Questioning data saturation as a useful concept for thematic analysis and sample-size rationales. Qualitative Res sport Exerc health.

[29] Ministry of Housing, Communities and Local Government. *Indices of Deprivation 2015 Explorer*; Ministry of Housing, Communities and Local Government: London, UK, 2015. Available from: 2015 English IMD explorer (communities.gov.uk).

[30] Ministry of Housing, Communities and Local Government. *English indices of deprivation 2019*, Ministry of Housing, Communities and Local Government: London, UK. Available from: English indices of deprivation 2019: Postcode Lookup (opendatacommunities.org).

[31] Phillips SM, Summerbell C, Hesketh KR, Saxena S, Hillier-Brown FC (2022). Parental views on the acceptability and feasibility of Measurement Tools used to Assess Movement Behaviour of Pre-School Children: a qualitative study. Int J Environ Res Public Health.

[32] Chief Medical Officers. UK Chief Medical Officers’ Physical Activity Guidelines. 2019. Available from: Physical activity guidelines: UK Chief Medical Officers’ report - GOV.UK (www.gov.uk).

[33] Terwee CB, Mokkink LB, van Poppel MN, Chinapaw MJ, van Mechelen W, de Vet HC (2010). Qualitative attributes and measurement properties of physical activity questionnaires. Sports Med.

[34] Mokkink LB, De Vet HC, Prinsen CA, Patrick DL, Alonso J, Bouter LM (2018). COSMIN Risk of Bias checklist for systematic reviews of patient-reported outcome measures. Qual Life Res.

[35] Clarke V, Braun V. Successful qualitative research: a practical guide for beginners. Success Qualitative Res. 2013:1–400.

[36] Braun V, Clarke V (2006). Using thematic analysis in psychology. Qualitative Res Psychol.

[37] Janz KF, Broffitt B, Levy SM (2005). Validation evidence for the Netherlands physical activity questionnaire for young children: the Iowa Bone Development Study. Res Q Exerc Sport.

[38] Tikotzky L, Sadeh A (2001). Sleep patterns and sleep disruptions in kindergarten children. J Clin Child Psychol.

[39] Chaput JP, Colley RC, Aubert S, Carson V, Janssen I, Roberts KC (2017). Proportion of preschool-aged children meeting the canadian 24-Hour Movement Guidelines and associations with adiposity: results from the Canadian Health Measures Survey. BMC Public Health.

[40] Bingham DD, Collings PJ, Clemes SA, Costa S, Santorelli G, Griffiths P (2016). Reliability and validity of the early years physical activity questionnaire (EY-PAQ). Sports.

[41] Ohayon M, Wickwire EM, Hirshkowitz M, Albert SM, Avidan A, Daly FJ (2017). National Sleep Foundation’s sleep quality recommendations: first report. Sleep health.

[42] Meredith-Jones K, Galland B, Haszard J, Gray A, Sayers R, Hanna M, Taylor B, Taylor R (2019). Do young children consistently meet 24-h sleep and activity guidelines? A longitudinal analysis using actigraphy. Int J Obes.

[43] Owens JA, Spirito A, McGuinn M (2000). The children’s Sleep Habits Questionnaire (CSHQ): psychometric properties of a survey instrument for school-aged children. Sleep.

[44] Janssen X, Martin A, Hughes AR, Hill CM, Kotronoulas G, Hesketh KR. Associations of screen time, sedentary time and physical activity with sleep in under 5s: a systematic review and meta-analysis. Sleep Med Rev. 2020(49):101226. 10.1016/j.smrv.2019.101226 PMC7034412 31778942

[45] Reilly JJ, Hughes AR, Janssen X, Hesketh KR, Livingstone S, Hill C (2020). GRADE-ADOLOPMENT process to develop 24-hour movement behavior recommendations and physical activity guidelines for the under 5s in the United Kingdom, 2019. J Phys Activity Health.

[46] Chia M, Tay LY, Chua TBK. The development of an online surveillance of digital media use in early childhood questionnaire-SMALLQ™- for Singapore. 2019.

[47] Hinkley T, Salmon J, Okely AD, Crawford D, Hesketh K (2012). The HAPPY study: development and reliability of a parent survey to assess correlates of preschool children’s physical activity. J Sci Med Sport.

[48] Townsend MS, Shilts M, Ontai L, Leavens L, Davidson C, Sitnick S. Obesity risk for young children: development and initial validation of an assessment tool for participants of federal nutrition programs. 2021.

[49] Adams J (2010). Prevalence and socio-demographic correlates of “active transport” in the UK: analysis of the UK time use survey 2005. Prev Med.

[50] Clifford Astbury C, Foley L, Penney TL, Adams J (2020). How does time use differ between individuals who do more versus less foodwork? A compositional data analysis of time use in the United Kingdom time use survey 2014–2015. Nutrients.

[51] Flesch Kincaid Calculator (2022). Flesch Kincaid Calculator.

[52] A Better Start. Better Start Bradford. 2021. Available from: https://www.betterstartbradford.org.uk/.

[53] HENRY. About Henry. 2020. Available from: https://www.henry.org.uk/about.

[54] Stuber JM, Middel CN, Mackenbach JD, Beulens JW, Lakerveld J (2020). Successfully recruiting adults with a low socioeconomic position into community-based lifestyle programs: a qualitative study on expert opinions. Int J Environ Res Public Health.

[55] Office for National Statistics. *Population denominators by broad ethnic group and for White British, local authorities in England and Wales: 2011 to 2019* Available at: https://www.ons.gov.uk/peoplepopulationandcommunity/populationandmigration/populationestimates/adhocs/008781populationdenominatorsbybroadethnicgroupandforwhitebritishlocalauthoritiesinenglandandwales2011to2017.

[56] Hidding LM, Chinapaw MJ, Belmon LS, Altenburg TM (2020). Co-creating a 24-hour movement behavior tool together with 9–12-year-old children using mixed-methods: MyDailyMoves. Int J Behav Nutr Phys Activity.

