# Supplementary material for: Co-design and content validity of the movement measurement in the early years (MoveMEY) tool for assessing movement behaviour of pre-school aged children
Source: Int J Behav Nutr Phys Act. 2023 Aug 4;20:95. doi: 10.1186/s12966-023-01486-2 (PMC10401865; doi:10.1186/s12966-023-01486-2)
Supplement: Supplementary file 2 — Additional file 2: Initial MoveMEY tool [file 12966_2023_1486_MOESM2_ESM.pdf]

Additional File 2: Initial MoveMEY tool

Measurement of Movement in the Early Years

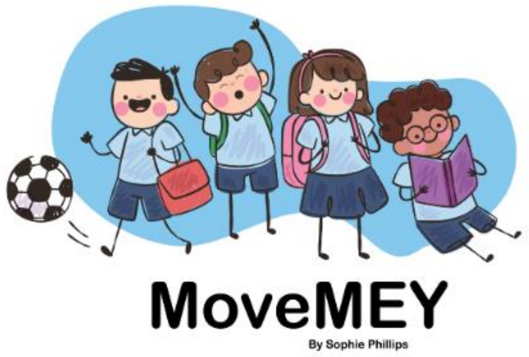

This questionnaire asks about the movement behaviours of your pre-school child.

There are three parts to this questionnaire:

Part 1: **Physical activity**- Bodily movement, including when your child is engaged in active play.

Part 2: **Sedentary behaviour-** When your child is sitting, reclining or lying.

Part 3: **Sleep**

Please answer all questions as accurately as possible. There are no right or wrong answers and all responses are confidential.

Durham University
fuse
NIHR | School for Public Health Research

Part 1- Physical Activity

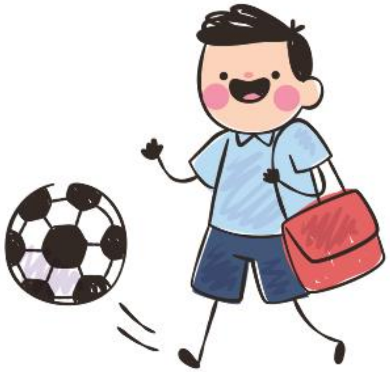

1a. In the past week, how many days and for how long each day would you say your child has spent actively playing outdoors (e.g., playing in the garden/yard, playing at the park, riding a bike, jumping on trampoline, climbing, skipping, throw/catch, football sessions)?

Please answer including outdoor play at home, at preschool/nursery or part of organised sessions.

How often	For how long each day (Please tick one box or write a time if more than one hour daily)				
Number of days this week.	Up to 15 mins	16-30 minutes	31-45 minutes	46-60 minutes	More than an hour per day. Please estimate time.
____/7					Hours: ____<br>Mins: ____

1b. Did any of this activity/active play make your child sweat or breathe harder? (Please circle)

Yes	No (Please proceed to Q2)

1c. If yes, how many hours/minutes of this activity/active play made your child sweat or breathe harder?

Hours: _____ Minutes: _____

2a. In the past week, how many days and for how long each day would you say your child has spent actively playing indoors (e.g. messy play, dancing, running around, rough and tumble play, sit and ride/push toys, indoor play area, active computer games such as Wii Sports, gymnastics/swimming classes)?

Please answer including indoor play at home, at preschool/nursery or part of organised sessions.

How often	For how long each day (Please tick one box or write a time if more than one hour daily)				
Number of days this week	Up to 15 mins	16-30 minutes	31-45 minutes	46-60 minutes	More than an hour per day. Please estimate time.
____/7					Hours:____<br>Mins:____

2b. Did any of this activity/active play make your child sweat or breathe harder? (Please circle)

Yes	No (Please proceed to Q3)

2c. If yes, how many hours/minutes of this activity/active play made your child sweat or breathe harder?

Hours: _____ Minutes:_____

3a. In the past week, how many days and for how long each day would you say your child has spent travelling by walking or cycling (e.g. to and from school)?

How often	For how long each day<br>(Please tick one box or write a time if more than one hour daily)				
Number of days this week	Up to 15 mins	16-30 minutes	31-45 minutes	46-60 minutes	More than an hour per day. Please estimate time.
____/7					Hours:____<br>Mins:____

3b. Did any of this time spent travelling make your child sweat or breathe harder? (Please circle)

Yes	No (Please proceed to Q4)

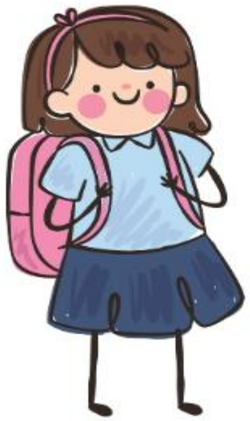

3c. If yes, how many hours/minutes of this time spent travelling made your child sweat or breathe harder?

Hours: _____   Minutes: _____

Part 2- Sedentary Behaviour

4. In the past week, how many days and for how long each day would you say your child has spent: in their pushchair, being carried, sat in the car/on public transport?

How often	For how long each day (Please tick one box or write a time if more than one hour daily)				
Number of days this week	Up to 15 mins	16-30 minutes	31-45 minutes	46-60 minutes	More than an hour per day. Please estimate time.
____/7					Hours:____<br>Mins:____

5. In the past week, how many days and for how long each day would you say your child has spent engaged in screen based activities (e.g watching TV, using a computer, tablet or mobile phone, playing computer games) whilst sitting, reclining or lying?

Please answer including both time at home and at preschool/nursery.

How often	For how long each day (Please tick one box or write a time if more than one hour daily)				
Number of days this week	Up to 15 mins	16-30 minutes	31-45 minutes	46-60 minutes	More than an hour per day. Please estimate time.
____/7					Hours:____<br>Mins:____

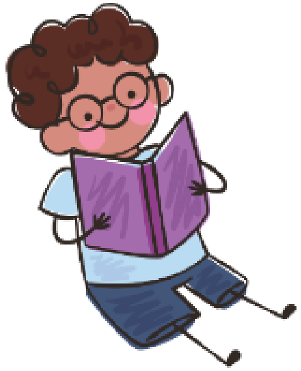

6. In the past week, how many days and for how long each day would you say your child has spent engaged in other activities (e.g reading, storytelling, colouring, playing with toys), whilst sitting, reclining or lying?

Please answer including both time at home and at preschool/nursery.

How often	For how long each day (Please tick one box or write a time if more than one hour daily)				
Number of days this week	Up to 15 mins	16-30 minutes	31-45 minutes	46-60 minutes	More than an hour per day. Please estimate time.
____/7					Hours:____<br>Mins:____

Part 3- Sleep

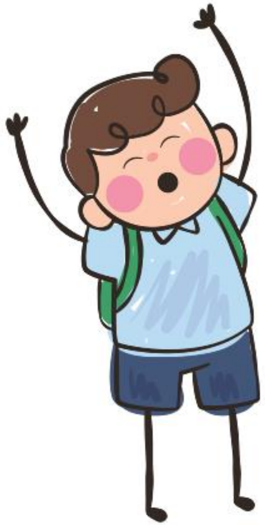

7a. Please write your child's usual bed time and wake up time.

Bed time: ________:________am/pm

Wake-up time: ________:________am/pm

7b. On which days of the week is this usually the case? (Please circle)

Monday	Tuesday	Wednesday	Thursday	Friday	Saturday	Sunday

8. On average, from the time that your child goes to bed, how long does it take them to fall asleep?

Minutes: __________

9. On average, how many times and for how long each time does your child wake up during their night time sleep?

How often	For how long each time (Please tick one box or write a time if more than one hour)				
Number of times per night	Up to 15 mins	16-30 minutes	31-45 minutes	46-60 minutes	More than an hour. Please estimate time.
					Hours:____<br>Mins:____

10. On average, how many times and for how long each time does your child nap during the day?

How often	For how long each time<br>(Please tick one box or write a time if more than one hour)				
Number of times per day	Up to 15 mins	16-30 minutes	31-45 minutes	46-60 minutes	More than an hour. Please estimate time.
					Hours:_____<br>Mins:_____

Thank you for completing this questionnaire.

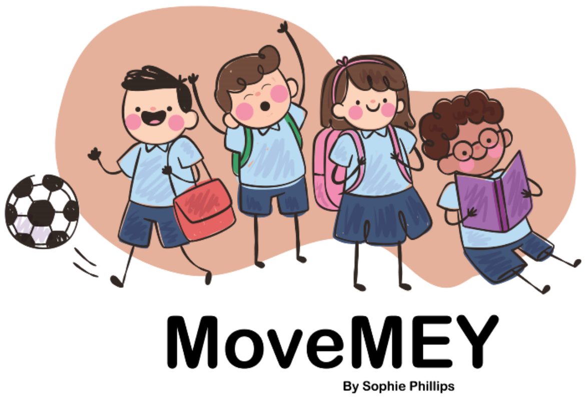

Additional File 1: Initial MoveMEY Scoring Sheet

Provisional Scoring System for MoveMEY Questionnaire

Behaviour/Construct	Questions used for scoring	Additional Comments
**Physical Activity**		
**Total Daily Physical Activity**	Q1a + Q2a+ Q3a	• Daily PA for each question calculated by: duration of activity each day (e.g 16 mins) multiplied by the frequency that activity occurred (e.g 5 days), this will be divided by 7 to estimate daily minutes of PA.<br>• The 'daily PA' time for all 3 individual questions will be summed to provide a **total daily PA.**<br>• For the calculation of PA, minimum reported durations will be used (e.g 16 mins), unless free text responses (exceeding 60 min/per) are used.
**Total Daily MVPA**	Q1c + Q2c + Q3c	• Total of the 3 questions/7
**Sedentary Time**		
**Total Daily Sedentary Time**	Q4 + Q5+ Q6	• Daily ST for each question calculated by: duration of activity each day (e.g 45 mins) multiplied by the frequency that activity occurred (e.g 5 days), this will be divided by 7 to estimate daily minutes of ST.<br>• The 'daily ST' for all 3 individual questions will be summed to provide a **total daily ST.**

		• For the calculation of ST, highest reported durations will be used (e.g 45 mins), unless free text responses (exceeding 60 min/per) are used.
**Total Daily Screen Time**	Q5	Duration of activity each day (e.g 45 mins) multiplied by the frequency that activity occurred (e.g 5 days), this will be divided by 7 to estimate daily screen time.
**Not being restrained for more than 1 hour**	Q4	Duration of activity each day (e.g 45 mins) multiplied by the frequency that activity occurred (e.g 5 days), this will be divided by 7 to estimate daily time restrained.
**When sedentary engaging in pursuits such as storytelling/reading**	Q6	Duration of activity each day (e.g 45 mins) multiplied by the frequency that activity occurred (e.g 5 days), this will be divided by 7 to estimate daily sedentary time engaged in non-screen based activities.
Sleep		
**10-13 hrs good quality sleep (may include a nap)**	Q7a - Q8 - Q9 + Q10	Total mins from time in bed (Q7a) – total mins to fall asleep – total mins awake during night + total nap duration
**Good quality sleep**	Time in bed= Q7a<br>Total sleep time=Q7a- Q8 - Q9	• Based on sleep efficiency – ratio of total sleep time to time in bed.<br>• ≥85% = good sleep quality<br>• Total sleep time (total time in bed – sleep latency – night time wakings)/Time in bed x 100
**Consistent wake and bed time**	Q7b	≥5 days

Additional File 1: Initial mapping of MoveMEY questions to guidelines

Guidelines		Provisional questions		
Physical Activity 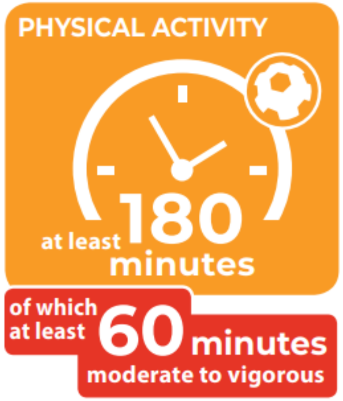	Spend at least 180 minutes in a variety of types of physical activities at any intensity.	In the past week, how many days and for how long each day would you say your child has spent actively playing outdoors (e.g. playing in the garden/yard, playing at the park, riding a bike, jumping on trampoline, climbing, skipping, throw/catch, football sessions)?	In the past week, how many days and for how long each day would you say your child has spent actively playing indoors (e.g. messy play, dancing, running around, rough and tumble play, sit and ride/push toys, indoor play area, active computer games such as Wii Sports, gymnastics/swimming classes)?	In the past week, how many days and for how long each day would you say your child has spent travelling by walking or cycling (e.g. to and from school)?
	At least 60 minutes is moderate- to vigorous intensity PA,	Did any of this activity/active play make your child sweat or breathe harder? (Please circle)	Did any of this activity/active play make your child sweat or breathe harder? (Please circle)	Did any of this activity/active play make your child sweat or breathe harder? (Please circle)
	At least 60 minutes is moderate- to vigorous intensity PA,	If yes, how many hours/minutes of this activity/active play made your child sweat or breathe harder?	If yes, how many hours/minutes of this activity/active play made your child sweat or breathe harder?	If yes, how many hours/minutes of this time spent travelling made your child sweat or breathe harder?
	Not be restrained for more than 1 hour at a time (e.g. prams/ strollers) or sit for	In the past week, how many days and for how long each day would you say your child has spent: in their pushchair, being		

Sedentary Behaviour<br>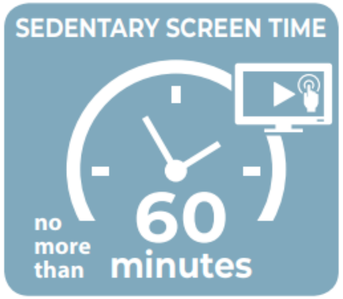	extended periods of time	carried, sat in the car/on public transport?		
	Sedentary screen time should be no more than 1 hour; less is better	In the past week, how many days and for how long each day would you say your child has spent engaged in screen based activities (e.g watching TV, using a computer, tablet or mobile phone, playing computer games) whilst sitting, reclining or lying?		
	When sedentary, engaging in reading and storytelling with a caregiver is encouraged	In the past week, how many days and for how long each day would you say your child has spent engaged in other quiet? activities (e.g reading, storytelling, colouring, playing with toys), whilst sitting, reclining or lying?		

Sleep 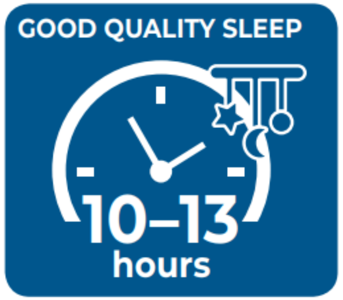	Have 10–13 hours of good quality sleep, which may include a nap	Please write your child's usual bed time and wake up time.	On average, from the time that your child goes to bed, how long does it take them to fall asleep?	On average, how many times and for how long each time does your child wake up during their night time sleep?<br><br>On average, how many times and for how long each time does your child nap during the day?
	Regular sleep and wake-up times	On which days of the week is this usually the case? (Please circle)		

Images extracted from WHO (2019).
