# Supplementary material for: Co-design and content validity of the movement measurement in the early years (MoveMEY) tool for assessing movement behaviour of pre-school aged children
Source: Int J Behav Nutr Phys Act. 2023 Aug 4;20:95. doi: 10.1186/s12966-023-01486-2 (PMC10401865; doi:10.1186/s12966-023-01486-2)
Supplement: Supplementary file 4 — Additional file 4: Final developed version of MoveMEY [file 12966_2023_1486_MOESM4_ESM.pdf]

Participant Number ________

Additional File 4: Final developed version of MoveMEY

Movement Measurement in the Early Years

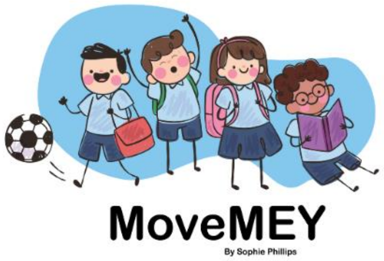

Diary for the caregivers of a child aged 3 or 4 years old. This diary asks about the movement behaviours that make up the 24 hours of your child's day.

There are four parts to this diary:

Part 1: **General questions –** Your child's health and if they have any disabilities.

Part 2: **Physical activity**- Any bodily movement, including when your child is engaged in active play or is actively travelling.

Part 3: **Sedentary behaviour-** Activities when your child is sitting, reclining or lying whilst awake.

Part 4: **Sleep-** The time your child spends asleep, during the day and at night.

Please complete this diary over the next 7 days. Please answer all questions as accurately as possible and try not to count the same activity more than once. Please put a 0 if the activity did not happen. There are no right or wrong answers and all responses are confidential.

Durham University

fuse

NIHR | School for Public Health Research

Part 1: General Questions

1. Does your child have any physical, neurodevelopmental, or medical condition or disability that affects their ability to play and be physically active?

Yes	No (Please proceed to Q2)

If yes, please state the condition or disability ____

2. Does your child have any medical sleep problems, such as night terrors?

Yes	No (Please proceed to Q3)

If yes, please state the sleep problem: ____

3. Is your child currently suffering from an illness, unwell or poorly (short or long term) that may affect their normal behaviours, including being active, movement, sitting or sleep?

Yes	No (Please proceed to Q4a)

If yes, please state the illness: ____

Part 2- Physical Activity

Part 2 of the diary is looking at the physical activity of your child, this is separated into different sections:

- **Activity whilst outdoors**
- **Activity used as a method of transport**
- **Activity whilst indoors**

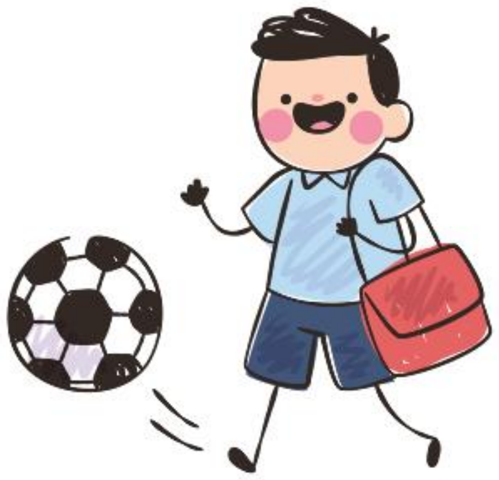

Note: Some questions ask for activity at '**home'** and '**nursery'**, by this we mean:

- **Home** – Any time when your child is not at nursery e.g. under parental/primary caregiver's care, at a friend or relatives house
- **Nursery**- When your child is at pre-school, nursery, playgroup, in childcare, childminders etc.

4a. Please state how many hours and minutes your child spends actively playing **outdoors** in each of the following (activities may include: running around, jumping on a trampoline, climbing, skipping, throw/catch).

	**Monday**		**Tuesday**		**Wednesday**		**Thursday**		**Friday**		**Saturday**		**Sunday**	
	**Home**	**Nursery**	**Home**	**Nursery**	**Home**	**Nursery**	**Home**	**Nursery**	**Home**	**Nursery**	**Home**	**Nursery**	**Home**	**Nursery**
	Total hours/minutes		Total hours/minutes		Total hours/minutes		Total hours/minutes		Total hours/minutes		Total hours/minutes		Total hours/minutes	
Example	*1 hour, 15 minutes*	*3 hours*	*1 hour*	*2 hours 30 minutes*	*2 hours 40 minutes*	*0*	*50 minutes*	*2 hours 20 minutes*	*2 hours 20 minutes*	*0*	*3 hours 15 minutes*	*0*	*1 hour*	*0*
Outdoor play at nursery														
Playing in the garden/yard														
Playing in the street														
Playing in a park / playground														
Playing in open outdoor spaces (e.g. beach, forest)														
Sport sessions (e.g football)														

Out for a walk/cycle (**not for travel**- see Q5a)														
Other activity (please specify):														
Other activity (please specify):														

4b. Did any of these activities make your child ‘huff and puff’, breathe harder, breathless, hot and sweaty, or need a drink or rest (Please circle).

Yes	No (Please proceed to Q5)

4c. If yes, please state how many hours/minutes of this activity made your child ‘huff and puff’, breathe harder, breathless, hot and sweaty, or stop the activity to ask for a drink or rest.

	**Monday**	**Tuesday**	**Wednesday**	**Thursday**	**Friday**	**Saturday**	**Sunday**
Total hours/minutes							

5a. Please state how many hours and minutes your child spends **actively travelling,** which could include travelling for leisure (e.g. to/from school, the shops, the park) each day.

	Monday	Tuesday	Wednesday	Thursday	Friday	Saturday	Sunday
	Total hours/minutes	Total hours/minutes	Total hours/minutes	Total hours/minutes	Total hours/minutes	Total hours/minutes	Total hours/minutes
Walking							
Cycling							
Scooter							
Other means of active transport (please specify):							

5b. Did any of these activities make your child 'huff and puff', breathe harder, breathless, hot and sweaty, or stop the activity to ask for a drink or rest (please circle).

Yes	No (Please proceed to Q6)

5c. If yes, please state how many hours/minutes of this activity made your child 'huff and puff', breathe harder, breathless, hot and sweaty, or stop the activity to ask for a drink or rest.

	Monday	Tuesday	Wednesday	Thursday	Friday	Saturday	Sunday
Total hours/minutes							

6a. Please state how many hours and minutes your child spends actively playing **indoors** (activities may include: dancing, running around, rough and tumble play, sit and ride push toys).

	**Monday**	**Tuesday**	**Wednesday**	**Thursday**	**Friday**	**Saturday**	**Sunday**
	Total hours/minutes	Total hours/minutes	Total hours/minutes	Total hours/minutes	Total hours/minutes	Total hours/minutes	Total hours/minutes
Playing actively in the home (**including** physically active video games e.g. Wii Sports or online exercise videos e.g. yoga, Zumba)							
Playing actively indoors at nursery							
Indoor play areas (e.g. soft play)							
Sport sessions (e.g. swimming, dance, motor skill classes)							
Other activity (please specify):							
Other activity (please specify):							

6b. Did any of these activities make your child 'huff and puff', breathe harder, breathless, hot and sweaty, or stop the activity to ask for a drink or rest. E.g. at soft play (Please circle)

Yes	No (Please proceed to Q7)

6c. If yes, please state how many hours/minutes of this activity made your child 'huff and puff', breathe harder, breathless, hot and sweaty, or stop the activity to ask for a drink or rest.

	**Monday**	**Tuesday**	**Wednesday**	**Thursday**	**Friday**	**Saturday**	**Sunday**
Total hours/minutes							

**Any additional comments for this section on physical activity:**

Part 3- Sedentary Behaviour

Part 3 of the diary is looking at the sedentary behaviour (activities when your child is sitting, lying or reclining whilst awake) of your child. This is separated into different sections:

- **Screen based activities**
- **Other sedentary activities**
- **Sedentary when travelling**

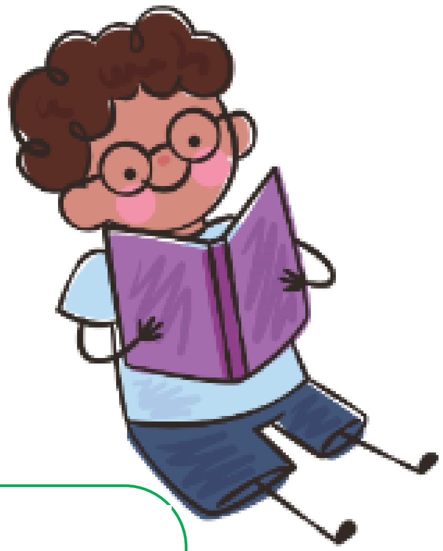

Note: Some questions ask for activity at '**home'** and '**nursery'**, by this we mean:

- **Home** – Any time when your child is not at school e.g. under parental/primary caregiver's care, at a friend or relatives house
- **Nursery**- When your child is at preschool, nursery, playgroup, in childcare, childminders etc.

Participant Number ________

7. Please state how long your child spends in **screen based** activities whilst in a sitting, reclining or lying position.

	**Monday**		**Tuesday**		**Wednesday**		**Thursday**		**Friday**		**Saturday**		**Sunday**	
	**Home**	**Nursery**	**Home**	**Nursery**	**Home**	**Nursery**	**Home**	**Nursery**	**Home**	**Nursery**	**Home**	**Nursery**	**Home**	**Nursery**
	Total hours/minutes		Total hours/minutes		Total hours/minutes		Total hours/minutes		Total hours/minutes		Total hours/minutes		Total hours/minutes	
Watching TV														
Using a computer/laptop														
Using a tablet/mobile phone														
Playing on a games console (e.g. PlayStation- **not** physically active games- see Q6a)														
Other (please specify):														

8. Please state at what time your child last uses a screen before going to bed (e.g. if child watches a film before bed).

	Example	**Monday**	**Tuesday**	**Wednesday**	**Thursday**	**Friday**	**Saturday**	**Sunday**
Time	7.15pm							

9. Please state how long your child spends **playing and in other activities** whilst sitting, reclining or lying, including **quiet or carpet time**.

	**Monday**		**Tuesday**		**Wednesday**		**Thursday**		**Friday**		**Saturday**		**Sunday**	
	**Home**	**Nursery**	**Home**	**Nursery**	**Home**	**Nursery**	**Home**	**Nursery**	**Home**	**Nursery**	**Home**	**Nursery**	**Home**	**Nursery**
	Total hours/minutes		Total hours/minutes		Total hours/minutes		Total hours/minutes		Total hours/minutes		Total hours/minutes		Total hours/minutes	
Playing with toys (including jigsaws/puzzles)														
Crafts (including colouring, painting, Play-Doh)														
Reading, storytelling, phonics														
Bath time														
Sat on the toilet/potty														
Sitting whilst eating														
Other (please specify):														
Other (please specify):														

10. Please state how long your child spends **seated whilst travelling**.

	Monday	Tuesday	Wednesday	Thursday	Friday	Saturday	Sunday
	Total hours/minutes	Total hours/minutes	Total hours/minutes	Total hours/minutes	Total hours/minutes	Total hours/minutes	Total hours/minutes
In the pushchair							
Being carried							
Sat in a car							
Sat on the bus/train/tram							
Other (please specify)							

**Any additional comments for this section on sedentary behaviour:**

Part 4- Sleep

Part 4 of the diary is looking at the sleep of your child, this is separated into different sections:

- **Bed time, sleep time, wake time, out of bed time**
- **Night time waking**
- **Day time sleep**

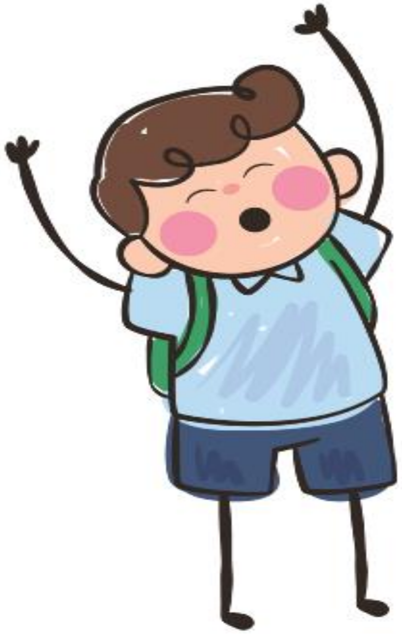

11. Please write the time your child goes to bed, the time they fall asleep, the time they wake up, and the time they get out of bed each day.

	Example	**Monday**	**Tuesday**	**Wednesday**	**Thursday**	**Friday**	**Saturday**	**Sunday**
**Bed time** (Time your child goes to bed)	*7.00pm*							
**Sleep time** (Time your child falls asleep)	*7.15pm*							
**Morning wake time** (Time your child wakes up)	*7.00am*							
**Out of bed time** (Time your child gets out of bed after their wake up time)	*7.05am*							

12. Please state how many times and for how long each time, that your child wakes up during their night time sleep.

	Example	**Monday**	**Tuesday**	**Wednesday**	**Thursday**	**Friday**	**Saturday**	**Sunday**
How many times	*2*							
How long each time	*20 minutes*							

13. Please state how many times and for how long each time that your child naps during the day.

	Example	**Monday**	**Tuesday**	**Wednesday**	**Thursday**	**Friday**	**Saturday**	**Sunday**
How many times	*1*							
How long each time	*45 minutes*							

**Any additional comments for this section on sleep:**

MoveMEY

Thank you for completing this diary!

MoveMEY

Additional File 3: Scoring Sheet for final developed version of MoveMEY

Scoring System for MoveMEY Measurement Tool

MoveMEY

Behaviour/Construct	Questions used for scoring	Additional Comments
General questions	1, 2, 3	General questions to be used as screening questions. If the answer is 'yes' to any of these questions, then child may be excluded or reported separately.
Physical Activity		
**Total Daily Physical Activity (PA)**	Q4a + Q5a+ Q6a	• Daily PA for each question calculated by sum of all hours and minutes of activity recorded each day.<br>• The 'daily PA' time for all 3 individual questions will be summed to provide a **total daily PA.**<br>• From this, will be able to determine how many days children are physically active and their average physical activity on those days (to establish if they meet the recommendation).
**Total Daily Moderate to Vigorous Physical Activity (MVPA)**	Q4c + Q5c + Q6c	• Daily MVPA calculated by the sum of the 3 questions.<br>• From this, we can determine how many days children engage in MVPA and their average MVPA activity on those days

		(to establish if they meet the recommendation).
Sedentary Behaviour		
**Total Daily Sedentary Behaviour (SB)**	Q7 + Q9+ Q10	• Daily SB for each question calculated by sum of hours and minutes spent in each of the activities each day.<br>• The 'daily SB' for all 3 individual questions will be summed to provide a **total daily SB.**<br>• From this, will be able to determine average SB of each day.
**Total Daily Screen Time**	Q7	• Sum of duration of hours and minutes spent in each activity, each day.<br>• From this, can determine how many days children engage in screen time and average screen time use per day.
**Screen time before bed**	Q8 (time), Q11 (bedtime)	• Hours/minutes difference between the time at which child last uses a screen (Q8), and the time child goes to bed (Q11).<br>• From this can determine if/when children uses a screen in the 1 hour leading up to bedtime.
**Not being restrained for more than 1 hour**	Q10 (in the pushchair, sat in a car, 'other' where child would be restrained- please specify what activity this is).	• Sum of duration of hours and minutes recorded for child being: 1) in the pushchair; 2) sat in a car; 3) other (please

		specify)- if this includes an activity where child would usually be restrained (please specify what activity this is), each day.<br>• From this, we can determine when children are restrained and average time per day.
**When sedentary engaging in pursuits such as storytelling/reading (daily ST engaged in non-screen based) activities)**	Q9	• Sum of duration of hours and minutes spent in each activity, each day.<br>• From this, we will be able to determine how many days children spend engaged in non-screen based sedentary pursuits, and the average time spent on these per day.
**Sleep**		
**10-13 hrs good quality sleep (may include a nap)**	Q11 - Q12 + Q13	• Total hours/mins from sleep time to wake time – total mins awake during night (Q12) + total nap duration (Q13).<br>• From this, we will be able to determine the amount of time a child spends asleep in a 24 hr day (and from this, whether they meet the recommendation).
**Good quality sleep**	Total time in bed= Q11 bedtime to out of bedtime<br>Total sleep time = Q11 sleep time to wake time	• Total time in bed = total hours/minutes between bedtime and out of bedtime

	Q11 –Q12+Q13	• Total sleep time = total hours/mins between sleep time and wake time – night-time waking + nap time<br><br>• Calculated by total sleep time divided by total time in bed x 100<br><br>• 'Good sleep quality' will be based on sleep efficiency – ratio of total sleep time to time in bed, ≥85% = good sleep quality.
**Consistent sleep and wake time**	Q11	≥5 days for sleep time and morning wake time. Consistent classified as within 30 minutes.

Additional File 3: Final developed version of MoveMEY mapped to guidelines

Guidelines		Final questions			Scoring
Physical Activity 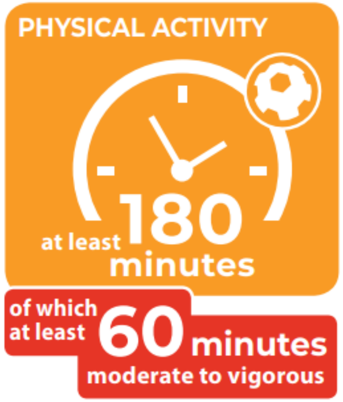	Spend at least 180 minutes in a variety of types of physical activities at any intensity.	Please state how many hours and minutes your child spends actively playing outdoors in each of the following (activities may include: running around, jumping on a trampoline, climbing, skipping, throw/catch).	Please state how many hours and minutes your child spends actively travelling, which could include travelling for leisure (e.g. to/from school, the shops, the park) each day.	Please state how many hours and minutes your child spends actively playing indoors (activities may include: dancing, running around, rough and tumble play, sit and ride push toys).	Daily PA for each question calculated by sum of all hours and minutes of activity recorded each day.<br><br>The 'daily PA' time for all 3 individual questions will be summed to provide a total daily PA.
	At least 60 minutes is moderate- to vigorous intensity PA	Did any of these activities make your child 'huff and puff', breathe harder, breathless, hot and sweaty, or stop the activity to ask for a drink. (Please circle)	Did any of these activities make your child 'huff and puff', breathe harder, breathless, hot and sweaty, or stop the activity to ask for a drink. (Please circle)	Did any of these activities make your child 'huff and puff', breathe harder, breathless, hot and sweaty, or stop the activity to ask for a drink. E.g. at	Daily moderate to vigorous intensity PA calculated by the sum of the 3 questions.

				soft play<br>(Please circle)	
	At least 60 minutes is moderate- to vigorous intensity PA.	If yes, please state how many hours/minutes of this activity made your child ‘huff and puff’, breathe harder, breathless, hot and sweaty, or stop the activity to ask for a drink.	If yes, please state how many hours/minutes of this activity made your child ‘huff and puff’, breathe harder, breathless, hot and sweaty, or stop the activity to ask for a drink.	If yes, please state how many hours/minutes of this activity made your child ‘huff and puff’, breathe harder, breathless, hot and sweaty, or stop the activity to ask for a drink.	
Sedentary Behaviour<br>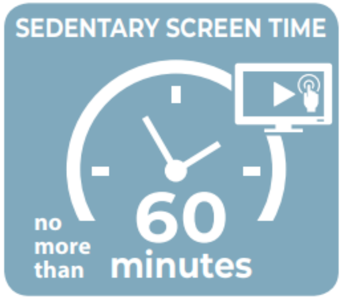 	Not be restrained for more than 1 hour at a time (e.g. prams/ strollers) or sit for extended periods of time	Please state how long your child spends seated whilst travelling. (Question includes rows for different types of travel e.g. in the pushchair, being carried, sat in a car).			Sum of duration of hours and minutes recorded for child being: 1) in the pushchair; 2) sat in a car; 3) other (please specify)- if this includes an activity where child would usually be restrained (please specify what activity

					this is), each day.
	Sedentary screen time should be no more than 1 hour; less is better	Please state how long your child spends in screen based activities whilst in a sitting, reclining or lying position.			Sum of duration of hours and minutes spent in each activity, each day.
	When sedentary, engaging in reading and storytelling with a caregiver is encouraged	Please state how long your child spends playing and in other activities whilst sitting, reclining or lying, including quiet or carpet time. (Question includes rows for different activities e.g. playing with toys, reading, storytelling, phonics).			Sum of duration of hours and minutes spent in each activity, each day.
Sleep 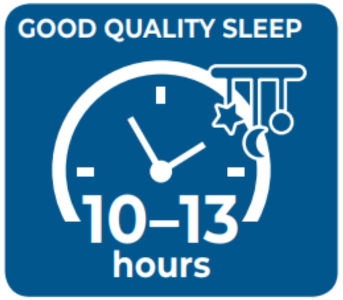	Have 10–13 hours of good quality sleep, which may include a nap	Please write the time your child goes to bed, the time they fall asleep, the time they wake up, and the time they get out of bed each day.	Please state how many times and for how long each time, that your child wakes up during their night time sleep.	Please state how many times and for how long each time that your child naps during the day.	Duration - Total hours/mins from sleep time to wake time minus total mins awake during night + total nap duration.<br><br>'Good quality' -

					Calculated by total sleep time (total hours/mins between sleep time and wake time minus night-time waking + nap time) divided by total time in bed (total hours/minutes between bedtime and out of bedtime) x 100<br><br>'Good sleep quality' will be based on sleep efficiency – ratio of total sleep time to time in bed, ≥85% = good sleep quality.
	Regular sleep and wake-up times	Please write the time your child goes to bed, the time they fall asleep, the time they			Consistent (classified as within 30 minutes) of

		wake up, and the time they get out of bed each day.			sleep and morning wake time for ≥5 days.
